# Long-term trends in the incidence of depressive disorders in China, the United States, India and globally: A comparative study from 1990 to 2019

**DOI:** 10.3389/fpsyg.2022.1066706

**Published:** 2023-01-17

**Authors:** Shuwen Wang, Tianhuan Lu, Jinyi Sun, Lihong Huang, Ruiqing Li, Tong Wang, Chuanhua Yu

**Affiliations:** School of Public Health, Wuhan University, Wuhan, China

**Keywords:** depressive disorders, incidence, Joinpoint regression, age-period-cohort analysis, trends

## Abstract

**Background:**

Depressive disorders have become an increasingly significant public health issue. This study is intended to show the trend of the incidence of depressive disorders in China, the United States, India and the world from 1990 to 2019, as well as the impact of age, period and cohort on it.

**Methods:**

Extracting incidence data from the Global Burden of Disease Study 2019, we determined trends in the age-standardized incidence rate (ASIR) using Joinpoint regression. An age-period-cohort analysis was implemented to describe the effects of age, period, and cohort, as well as the long-term tendencies.

**Results:**

From 1990 to 2019, the ASIR of depressive disorders in China was lower than that in the United States; India is lower than the United States in the first 5 years, showing a downward trend. The incidence in India and the United States is higher than the global average. The ASIR of women in the three countries is higher than that of men. In China, the elderly, early period and people born around 1954 have a higher risk of depressive disorders. In the United States, young people born around 1999 have a higher risk of depressive disorders. India is similar to China.

**Conclusion:**

From 1990 to 2019, the age effect of China as a whole increased, and the period became stable, and the cohort effect declined. The overall age and period effects of the United States reduced, while the cohort effect increased. The age effect in India increased, while the period and cohort effects decreased. Depressive disorders are becoming ever more serious worldwide, and we’d better take measures to reduce its incidence according to the cohort effect of each age group.

## 1. Introduction

With the rapid development of countries, the burden of non-communicable diseases (NCDs), including mental disorders, neoplasms, cardiovascular diseases, etc., is increasing (GBD 2017 Disease, Injury Incidence and Prevalence Collaborators, 2018).

Mental disorder is a condition manifested as cognitive disability, emotional regulation disorder or unusual behavior, which mainly reflects the underlying psychological dysfunction (Battle, 2013). Mental disorder has long been an important public health problem. Besides, with rapid socio-financial development, it has progressively become a major cause of the disease burden (Patel et al., 2018). However, until 1980, the burden of mental disorders was seriously undervalued, since mental disorders cause death only in rare cases and their burden of disease is measured mainly on the basis of indicators of mortality rather than morbidity (Murray et al., 1994). In the 1990 Global Burden of Disease Study (GBD 1990), nearly a quarter of all non-fatal burdens were related to neuropsychiatric disorders, at a time when mental, neurological and substance use disorders had become a public health’s biggest problem (Murray and Lopez, 1994). In addition, from 1990 to 2019, because of the main demographic, surroundings and sociopolitical transformations, particularly the alteration and diversification of social norms and values, an aging population growth and the increasing pressure of life, the global burden of mental disorders in all countries increased significantly, which poses a huge challenge to national mental health. (Fu et al., 2013; Charlson et al., 2016; Heim et al., 2017; Patel et al., 2018; Wang and Tapia Granados, 2019). More specifically, from 654.8 million (95%UI 603.6–708.1) cases in 1990 to 970.1 million (900.9–1044.4) cases in 2019, an increase of about 48.1% according to a Global Burden of Disease Study (GBD; GBD 2019 Mental Disorders Collaborators, 2022). Globally, mental disorders were the 13th leading cause of disability-adjusted life years (DALYs) in 1990 and the 7th leading cause in 2019. In 1990 and 2019, mental disorders were the second leading cause of disability among young people globally (GBD 2019 Mental Disorders Collaborators, 2022). The decreasing burden of infectious diseases between 1990 and 2019 has been offset by an increase in non-communicable diseases, including mental illness (GBD 2019 Diseases and Injuries Collaborators, 2020; GBD 2019 Risk Factors Collaborators, 2020a; GBD 2019 Demographics Collaborators, 2020b).

Depressive disorder is a worldwide shared mental disorder that has a serious impact on our physical and mental health. Symptoms mainly include low mood, negative attitude towards life, lack of passion and fighting spirit in life, insomnia, decreased quality of life, and suicidal tendency (Fava and Kendler, 2000; Nestler et al., 2002). Studies have shown that major depressive disorder increases the risk of death, approximately doubling it (Yu et al., 2016). Depressive disorders affect an estimated 5% of adults worldwide and significantly increases the global burden of disease (Organization WH, 2020). Previous studies have shown that depressive disorders ranked 13th among the top 25 causes of DALY in 2019, and second among the top 25 leading causes of youth disability (GBD 2019 Mental Disorders Collaborators, 2022). Among mental disorders, depressive disorders rank highest in all age groups except the 0–14 age group and are the main cause of burden (GBD 2019 Mental Disorders Collaborators, 2022).

Depressive disorder is the main cause of the burden of mental, neurological and substance use disorders worldwide, in both developed and developing regions (Charlson et al., 2016). In 2017, the DALYs of depressive disorders in China was 480.4/100,000 (95%UI: 339.8–653.6), in India was 572.8/100,000 (95%UI: 404.5–778.4), and in the United States was 736.9/100, 000 (95%UI: 521.7–993.3; Ren et al., 2020).

In recent years, as mental health has gradually come into public view, related research has also been carried out gradually. However, earlier studies have provided only a cursory analysis of trends in depressive disorders. There are few studies comparing trends in China, the US, India and globally. There are also few studies using the age-period-cohort analysis method on the incidence trend of depressive disorders, which can describe age, period and cohort effects, respectively. Therefore, we present the results of the GBD 2019 study, using age-period-cohort and Joinpoint analysis to assess the trend of depressive disorders incidence in China, the US and India and globally. Our study will help develop prevention strategies to reduce the incidence of depressive disorders.

## 2. Materials and methods

### 2.1. Data sources

The Global Burden of Diseases, Injuries, and Risk Factors Study 2019 (GBD, 2019) was provided by the Institute for Health Metrics and Evaluation (IHME; http://www.healthdata.org/)-an independent global health research center at the University of Washington. GBD 2019 uses data from a variety of sources, containing household surveys, censuses, essential statistics and civil registration, to calculate 286 causes of death, 369 diseases and injuries, and 87 risk factors roughly in 204 nations and districts. The GBD estimates the indicators of incidence, prevalence, deaths, years of life lost (YLLs), years lived with disability (YLDs), and disability-adjusted life years (DALYs) of each disease, which are reported by year, location, age and gender. GBD aims to establish comprehensive and comparable global health indicators (GBD 2019 Diseases and Injuries Collaborators, 2020). Depression in GBD includes major depressive disorder and dysthymia, and ICD 10 codes include F32-F33.9 and F34.1.

We used the Global Health Data Exchange (GHDx) to extract the total incidence of mental disorders and depressive disorders from 1990 to 2019. Age is mainly divided into the following age groups: 20–24 years old, 25–29 years old, 30–34 years old, 35–39 years old, … and 85–89. Countries and regions were selected as China, the United States, India and the global.

### 2.2. Statistical analysis

Using Joinpoint regression analysis, we estimated trends in the age-standardized incidence (ASIR) of mental disorders and depressive disorders over time. This model mainly uses logarithmic transformation of the rate and calculates the standard error according to binomial approximation (Kim et al., 2000). The model analysis can help us identify the point of change in each trend, which will help us determine the change over time in the incidence of mental disorders and depressive disorders in China, the United States and India and globally. Furthermore, we can calculate the annual percentage change (APC), average annual percentage change (AAPC), and 95% confidence interval (CI) for each trend in the model by performing a piecewise regression analysis (Clegg et al., 2009). We performed Joinpoint analysis by using the Joinpoint regression program version 4.9.0.1 (Information Management Services, Inc., Calverton, MD, USA) from the Statistical Research and Applications Branch of the Surveillance Research Program of the U.S. National Cancer Institute.

Age-period-cohort model is a log-linear regression model based on Poisson distribution. Its basic idea is to determine the value of age, period and cohort effect by fitting the regression relation between target event incidence and age, period and birth cohort. Age-period-cohort analysis is an analytical method that can simultaneously describe the impact of social development, historical change, demographic change and environmental factors on people. It eliminates the interaction between age, period and cohort effects, displaying the effect of age, period and cohort alone on incidence. In addition, due to the relationship of cohort = period-age (Yang et al., 2014), the Age-period-cohort model has some collinearity problems, which may have an impact on the consequences. Therefore, in order to eliminate collinearity, this study adopts the method based on the internal estimation (IE) algorithm, which has been proved to be estimable, non-bias, effective and asymptotic, and has certain feasibility. The model expression of age-period-cohort is


Y=log(M)=μ+αage1+βperiod1+γcohort1+ε

Where M is incidence rate, μ is intercept, α, β and γ are coefficient of age, period and cohort effects, respectively, and ε is random error.

This study extracted the incidence of mental disorders and depressive disorders in people aged 20–89 years from 1990 to 2019 in China, the United States and India and globally from GBD. We divided patients into 19 birth cohorts (1909–1913; 1914–1918; 1919–1923; 1924–1928; until 1995 to 1999). Patients were divided into five time periods (1994–1999, 1999–2004, 2004–2009, 2009–2014, and 2014–2019). The incidence of mental disorders was age-adjusted for 14 age groups (20–24 years, 25–29 years, 30–34 years, 35–39 years, 40–44 years, 45–49 years, 50–54 years, 55–59 years, 60–64 years, 65–69 years, 70–74 years, 75–79 years, 80–84 years and 85–89 years). According to the WHO (World Health Organization) report (Ahmad et al., 2001), the population is divided into young (age < 44 years), middle (age 45–59 years), and old (age > 60 years) age groups. The age-period-cohort model is mainly implemented by stata16.0 software. Bias, Akaike information criterion (AIC) and Bayesian information criterion (BIC) were used to evaluate the accuracy and degree of fit of the model. Standard Error (SE) coefficients and hazard ratios were calculated.

## 3. Results

### 3.1. Trend of mental disorders in China, the United States, India and the global from 1990 to 2019

**Figure 1:**
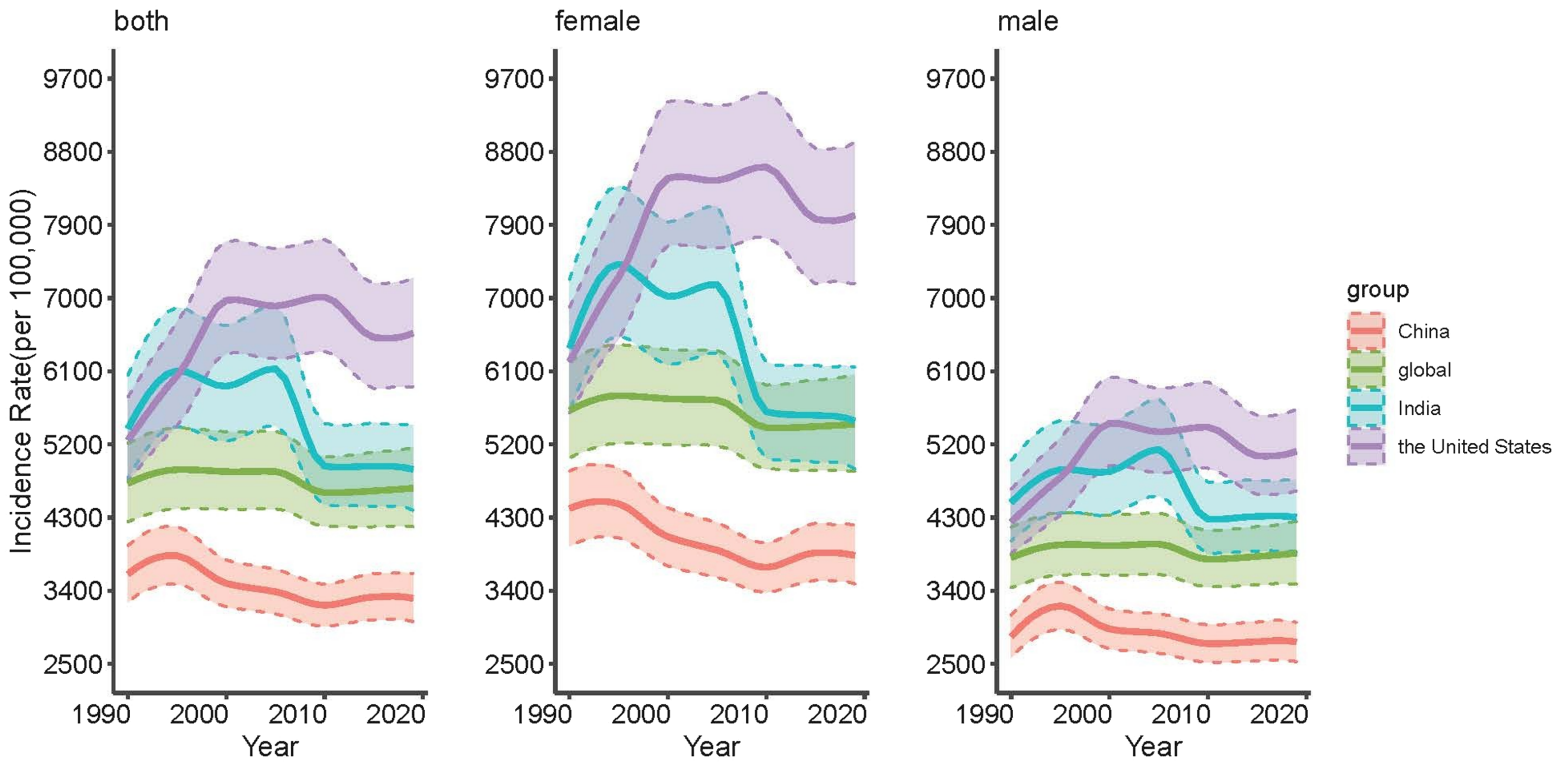
Trends of age-standardized incidence rates (ASIR) for mental disorders in the whole population, males and females in China, the United States, India and the global, 1990 to 2019.

Figure 1 shows the trend of mental disorders in China, the United States, India and the global from 1990 to 2019. From 1990 to 2019, the ASIR of American men and women showed an overall upward trend, of which the upward trend of women was more obvious, with an increase of 28.89%, and the ASIR of both men and women was higher than the global level; The ASIR of Chinese men and women tended to be stable or even slightly decreased, and both were lower than the global level; In India, the ASIR of men and women showed a significant downward trend from 2005 to 2010, but it was always higher than the global level; The global ASIR has not changed significantly in the past 30 years. In addition, compared with other countries, the ASIR of Chinese men and women with mental disorders was the lowest among the three countries, while the ASIR of the United States was the highest after 1995, and was lower than that of India between 1990 and 1995. It is worth noting that the ASIR of men with mental disorders in China, the United States, India and the world is lower than that of women. The exact values are shown in Supplementary Table S1.

### 3.2. Trend of depressive disorders in China, the United States, India and global from 1990 to 2019

**Figure 2:**
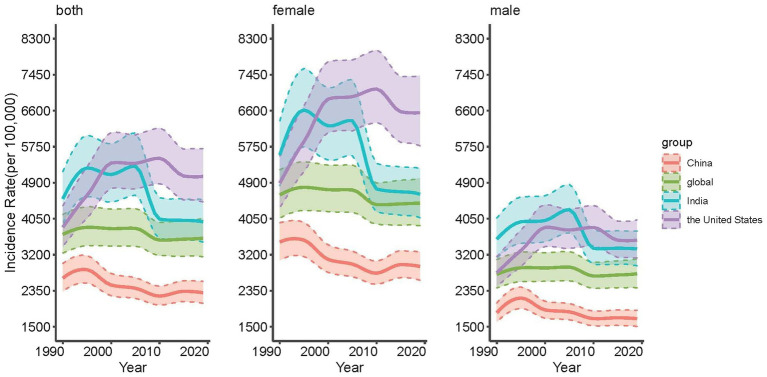
Trends of age-standardized incidence rates for depressive disorders in the whole population, males and females in China, the United States, India and the global, 1990 to 2019.

Figure 2 presents the trend of depressive disorders in China, the United States, India and the global from 1990 to 2019. It can be seen from the picture that the incidence of depressive disorders for females is higher than that for males in the three countries and the world. At the national level, the incidence of depressive disorders in China is the lowest and lower than the global level, and both India and the United States are higher than the global level. For the entire population, the incidence in India in the first 10 years is higher than that in the United States, and the opposite is true in the last two decades; Furthermore, as far as the trend is concerned, China and the world tend to be stable, India shows a significant downward tendency, and the United States shows a significant upward trend. The exact values are indicated in Supplementary Table S2.

### 3.3. Trends of age-standardized incidence rates for mental disorders and depressive disorders using Joinpoint regression analysis

#### 3.3.1. Trends of age-standardized incidence rates for mental disorders using Joinpoint regression analysis

Table 1 presents the APC and AAPC for the incidence of mental disorders in China, the United States, India, and globally from 1990 to 2019. From 1990 to 2019, the incidence of mental disorders in the whole population in China changed from 3604.93/100000 to 3305.02/100000, showing a general downward trend. The AAPC for the entire period was −0.3% (−0.5, −0.1%). The incidence rate of women showed a downward trend, and AAPC was −0.5% (−0.6, −0.4%). The male incidence rate increased significantly from 1990 to 1992 at a rate of 4.2% per year, with no significant change during the entire period. In the United States, from 1990 to 2019, the incidence of mental disorders in the whole population changed from 5,253.38 per 100,000 to 6568.54 per 100,000, showing an overall upward trend. The AAPC for the entire period was 0.8% (0.7, 0.9%). From 1990 to 2000, the incidence rate of women increased sharply at an annual rate of 3.2%, with an upward trend in the entire period. The incidence rate of males increased significantly from 1990 to 1999, with an average annual increase of 3.5% from 1996 to 1999, showing an increasing trend during the entire period. In India, from 1990 to 2019, the population incidence of mental disorders reduced from 5,393.32 per 100,000 to 4,893.47 per 100,000, showing an overall downward trend. The AAPC of the whole period was −0.3%(−0.4, −0.3%). From 2005 to 2010, there was a significant decrease in female incidence, with an average annual decrease of 5.1 percent, and a downward trend throughout the period. The overall trend of male incidence was the same as that of female incidence, with an average annual decrease of 3.8% from 2005 to 2010 and an AAPC of −0.1% (−0.2, −0.1%) during the entire period. Globally, from 1990 to 2019, the incidence of mental disorders of the whole population decreased from 4,720.70/100000 to 4,660.91/100000, showing an overall downward trend. The AAPC of the whole period is −0.1% (−0.1, −0.0%). The incidence rate of women was similar to that of the whole population, but the decreasing tendency was more obvious, with the AAPC of −0.2% (−0.3, −0.2%). There was no significant fluctuation in male incidence throughout the period.

**Table 1:** Trends of age-standardized incidence rates for mental disorders in the whole population, males and females in China, the United States, India and the global, 1990 to 2019.

Segments	Both		Female		Male	
	Year	APC (95% CI)	Year	APC (95% CI)	Year	APC (95% CI)
China						
Trend1	1990–1993	2.0* (1.2, 2.7)	1990–1995	0.3* (0.1, 0.4)	1990–1992	4.2* (3.5, 5.0)
Trend2	1993–1996	−0.2 (−1.7, 1.2)	1995–2000	−2.1* (−2.3, −1.9)	1992–1995	1.7* (0.9, 2.4)
Trend3	1996–1999	−2.4* (−3.7, −1.0)	2000–2005	−0.8* (−1.0, −0.6)	1995–2000	−2.0* (−2.3, −1.8)
Trend4	1999–2010	−0.8* (−0.9, −0.7)	2005–2010	−1.2* (−1.4, −1.0)	2000–2005	−0.3* (−0.5, −0.1)
Trend5	2010–2016	0.6* (0.3, 0.9)	2010–2015	1.1* (0.9, 1.3)	2005–2010	−0.9* (−1.1, −0.7)
Trend6	2016–2019	−0.3 (−1.0, 0.4)	2015–2019	−0.3* (−0.5, −0.0)	2010–2019	0.2* (0.1, 0.2)
AAPC	1990–2019	−0.3* (−0.5, −0.1)	1990–2019	−0.5* (−0.6, −0.4)	1990–2019	−0.1 (−0.2, 0.0)
The United States						
Trend1	1990–2000	2.9* (2.8, 3.0)	1990–2000	3.2* (3.1, 3.3)	1990–1996	2.5* (2.4, 2.5)
Trend2	2000–2005	−0.4* (−0.7, −0.1)	2000–2005	−0.3 (−0.6, 0.1)	1996–1999	3.5* (2.9, 4.0)
Trend3	2005–2010	0.4* (0.1, 0.8)	2005–2010	0.5* (0.2, 0.9)	1999–2006	−0.2* (−0.3, −0.2)
Trend4	2010–2015	−1.6* (−1.9, −1.3)	2010–2015	−1.7* (−2.0, −1.4)	2006–2010	0.3* (0.1, 0.6)
Trend5	2015–2019	0.2 (−0.1, 0.5)	2015–2019	0.2 (−0.2, 0.5)	2010–2015	−1.5* (−1.6, −1.3)
Trend6	-		-		2015–2019	0.3* (0.1, 0.5)
AAPC	1990–2019	0.8* (0.7, 0.9)	1990–2019	0.9* (0.8, 1.0)	1990–2019	0.6* (0.6, 0.7)
India						
Trend1	1990–1994	3.1* (2.8, 3.4)	1990–1994	3.8* (3.4, 4.1)	1990–1994	2.1* (1.9, 2.3)
Trend2	1994–2000	−0.7* (−0.9, −0.4)	1994–2001	−1.0* (−1.1, −0.8)	1994–2000	−0.1 (−0.3, 0.1)
Trend3	2000–2005	1.0* (0.7, 1.3)	2001–2005	1.0* (0.4, 1.5)	2000–2005	1.3* (1.1, 1.5)
Trend4	2005–2010	−4.5* (−4.8, −4.3)	2005–2010	−5.1* (−5.4–4.8)	2005–2010	−3.8* (−4.0, −3.6)
Trend5	2010–2019	0.0 (−0.1, 0.1)	2010–2019	−0.1* (−0.2, −0.0)	2010–2019	0.2* (0.1, 0.2)
Trend6	-		-		-	
AAPC	1990–2019	−0.3* (−0.4, −0.3)	1990–2019	−0.5* (−0.6, −0.4)	1990–2019	−0.1* (−0.2, −0.1)
Global						
Trend1	1990–1994	0.8* (0.7, 0.9)	1990–1994	0.7* (0.7, 0.8)	1990–1994	1.0* (0.9, 1.1)
Trend2	1994–2005	−0.0* (−0.1, −0.0)	1994–2005	−0.1* (−0.1, −0.1)	1994–2001	−0.0* (−0.1, −0.0)
Trend3	2005–2010	−1.1* (−1.2, −1.1)	2005–2010	−1.3* (−1.3, −1.2)	2001–2005	0.2* (0.0, 0.3)
Trend4	2010–2019	0.2* (0.1, 0.2)	2010–2019	−0.1* (0.1 0.1)	2005–2010	−1.0* (−1.1, −1.0)
Trend5	-		-		2010–2019	0.2* (0.2, 0.3)
Trend6	-		-		-	
AAPC	1990–2019	−0.1* (−0.1, −0.0)	1990–2019	−0.1* (−0.1, −0.1)	1990–2019	0.0* (0.0, 0.1)

APC, annual percentage change; AAPC, average annual percent change; CI, confidence interval.

* Indicates that the Annual Percent Change (APC) and the Average Annual Percent Change (AAPC) are significantly different from zero at the alpha = 0.05 level.

#### 3.3.2. Trends of age-standardized incidence rates for depressive disorders using Joinpoint regression analysis

Table 2 presents the APC and AAPC for depressive disorders incidence in China, the United States, India, and globally from 1990 to 2019. From 1990 to 2019, the incidence of depressive disorders in the entire population in China changed from 2,647.72/100000 to 2,301.41/100000, showing a general downward trend. The AAPC for the whole period was −0.5% (−0.7, −0.4%); the incidence rate of women showed a downward trend, AAPC was −0.7% (−0.7, −0.6%). The incidence rate of males increased significantly from 1990 to 1992 at an annual rate of 5.9%, with a downward trend throughout the period. In the United States, from 1990 to 2019, the incidence of depressive disorders in the whole population changed from 3849.98 per 100,000 to 5047.93 per 100,000, showing an overall upward trend. The AAPC for the whole period was 0.9% (0.8, 1.1%). From 1990 to 1999, the incidence rate of women increased sharply by 3.7% per year and displayed an upward trend throughout the period. The incidence rate of males increased significantly from 1990 to 1999 and showed an increasing trend throughout the period. In India, from 1990 to 2019, the population incidence of depressive disorders decreased from 4,520.76 per 100,000 to 3,975.68 per 100,000, showing an overall downward trend. The AAPC of the whole period was −0.5% (−0.5, −0.4%). From 2005 to 2010, there was a noteworthy reduce in female incidence, with an average annual decrease of 6.1 percent and a downward trend throughout the period. The incidence rate of males declined by 5.0% annually from 2005 to 2010. Globally, from 1990 to 2019, the incidence of depressive disorders in the national population decreased from 3,681.24/100000 to 3,588.25/100000, showing an overall downward trend. The AAPC of the entire period is −0.1% (−0.1, −0.1%). The incidence rate of women is similar to that of the whole population, but the declining trend is more obvious, AAPC is −0.2% (−0.2, −0.1%). There was no significant fluctuation in male incidence over the entire period.

**Table 2:** Trends of age-standardized incidence rates for depressive disorders in the whole population, males and females in China, the United States, India and the global, 1990 to 2019.

Segments	Both		Female		Male	
	Year	APC (95% CI)	Year	APC (95% CI)	Year	APC (95% CI)
China						
Trend1	1990–1995	1.5* (1.1, 1.8)	1990–1995	0.3* (0.1, 0.5)	1990–1992	5.9* (4.4, 7.5)
Trend2	1995–2000	−3.0* (−3.4, −2.7)	1995–2000	−3.0* (−3.2, −2.7)	1992–1995	2.3* (0.9, 3.8)
Trend3	2000–2005	−0.5* (−0.9, −0.1)	2000–2005	−0.7* (−0.9, −0.4)	1995–2000	−3.0* (−3.5, −2.6)
Trend4	2005–2010	−1.7* (−2.1, −1.4)	2005–2010	−1.6* (−1.8, −1.4)	2000–2005	−0.3 (−0.7, 0.1)
Trend5	2010–2015	1.2* (0.8, 1.6)	2010–2015	1.7* (1.4, 1.9)	2005–2009	−2.2* (−2.8, −1.6)
Trend6	2015–2019	−0.4* (−0.8, −0.0)	2015–2019	−0.5* (−0.8, −0.2)	2009–2019	0.0 (−0.1, 0.1)
AAPC	1990–2019	−0.5* (−0.7, −0.4)	1990–2019	−0.7* (−0.7, −0.6)	1990–2019	−0.3* (−0.5, −0.1)
The United States						
Trend1	1990–1999	3.6* (3.5, 3.7)	1990–1999	3.7* (3.6, 3.8)	1990–1999	3.6* (3.5, 3.7)
Trend2	1999–2002	0.4 (−0.5, 1.4)	1999–2006	0.3* (0.2, 0.5)	1999–2006	−0.1* (−0.2, 0.0)
Trend3	2002–2005	−0.2 (−1.1, 0.7)	2006–2010	0.6* (0.2, 1.1)	2006–2010	0.5* (0.1, 0.8)
Trend4	2005–2010	0.6* (0.3, 0.9)	2010–2015	−1.7* (−2.0, −1.4)	2010–2015	−1.8* (−2.0, −1.6)
Trend5	2010–2015	−1.7* (−2.0, −1.4)	2015–2019	−0.1 (−0.4, 0.2)	2015–2019	0.0 (−0.2, 0.3)
Trend6	2015–2019	−0.0 (−0.3, 0.3)	-		-	
AAPC	1990–2019	0.9* (0.8, 1.1)	1990–2019	1.0* (0.9, 1.1)	1990–2019	0.8* (0.8, 0.9)
India						
Trend1	1990–1994	3.7* (3.4, 4.0)	1990–1994	4.4* (4.0, 4.8)	1990–1994	2.6* (2.3, 2.8)
Trend2	1994–2000	−0.6* (−0.8, −0.4)	1994–2001	−1.0* (−1.2, −0.8)	1994–2000	0.1 (−0.1, 0.3)
Trend3	2000–2005	1.1* (0.8, 1.4)	2001–2005	1.0* (0.4, 1.7)	2000–2005	1.6* (1.3, 1.8)
Trend4	2005–2010	−5.6* (−5.9, −5.3)	2005–2010	−6.1* (−6.4–5.7)	2005–2010	−5.0* (−5.2–4.8)
Trend5	2010–2019	−0.1 (−0.2, 0.0)	2010–2019	−0.2* (−0.3, −0.1)	2010–2019	0.1 (−0.0, 0.1)
Trend6	-		-		-	
AAPC	1990–2019	−0.5* (−0.5, −0.4)	1990–2019	−0.7* (−0.8, −0.5)	1990–2019	−0.2* (−0.3, −0.2)
Global						
Trend1	1990–1994	1.1* (1.0, 1.2)	1990–1994	0.9* (0.7, 1.1)	1990–1994	1.3* (1.2, 1.4)
Trend2	1994–2001	−0.1* (−0.2, −0.1)	1994–2006	−0.2* (−0.2, −0.1)	1994–2001	−0.0 (−0.1, 0.1)
Trend3	2001–2005	0.1* (0.0, 0.3)	2006–2009	−2.2* (−2.7, −1.7)	2001–2005	0.2* (0.1, 0.4)
Trend4	2005–2010	−1.6* (−1.7, −1.5)	2009–2012	−0.2 (−0.7, −0.3)	2005–2010	−1.6* (−1.7, −1.5)
Trend5	2010–2019	0.2* (0.1, 0.2)	2012–2019	0.1* (0.1, 0.2)	2010–2019	0.2* (0.2, 0.3)
Trend6	-		-		-	
AAPC	1990–2019	−0.1* (−0.1, −0.1)	1990–2019	−0.2* (−0.2, −0.1)	1990–2019	0.0 (−0.0, 0.0)

APC, annual percentage change; AAPC, average annual percent change; CI, confidence interval.

* Indicates that the Annual Percent Change (APC) and the Average Annual Percent Change (AAPC) are significantly different from zero at the alpha = 0.05 level.

### 3.4. Age, period, and cohort changes in depressive disorders incidence

**Figure 3:**
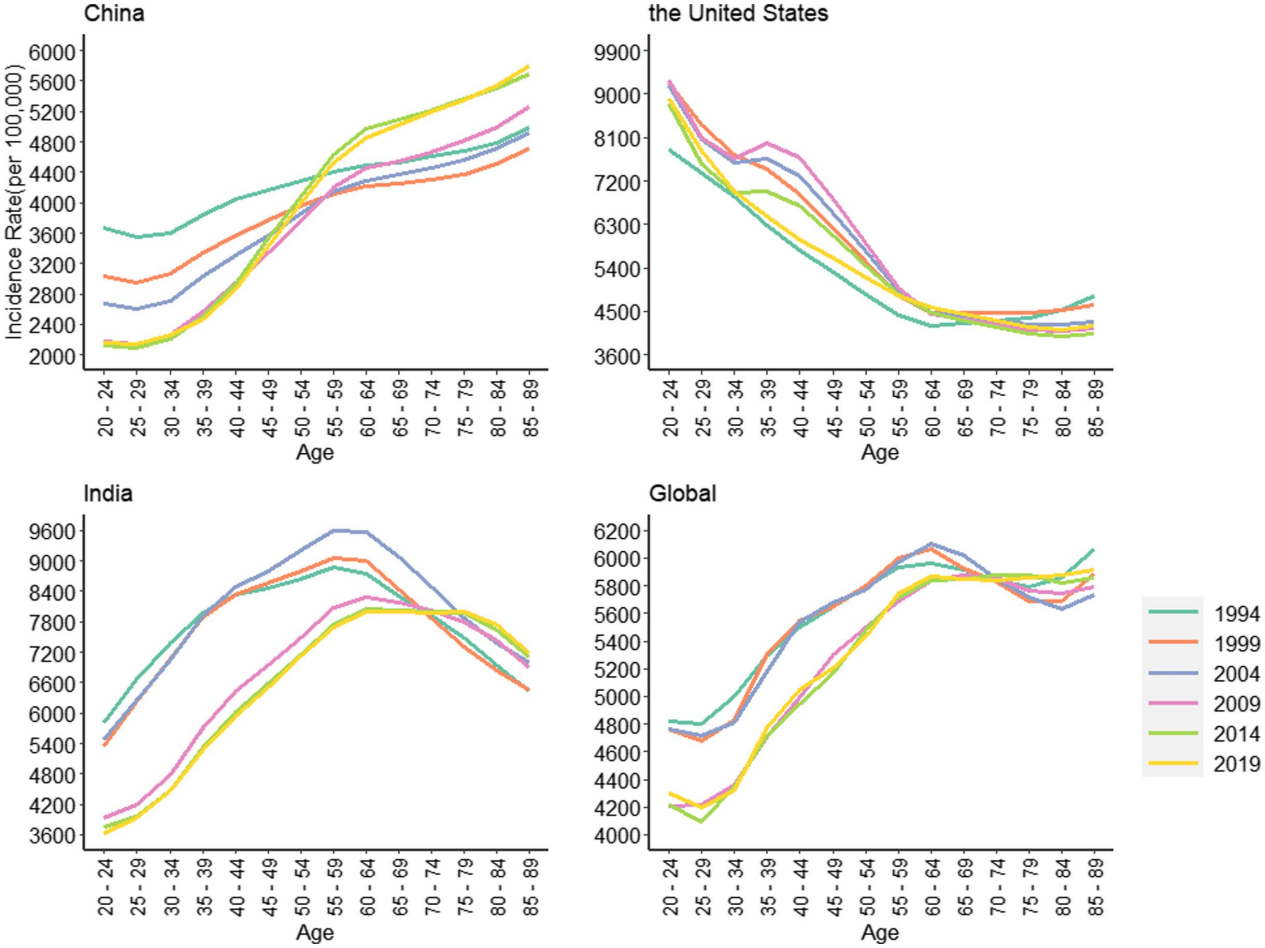
Trends of age-standardized incidence rates for depressive disorders in China, the United States, India and the global, 1990 to 2019.

Figure 3 shows the trends in the depressive disorders between different age groups from 1990 to 2019. As can be seen from the figure, the trend of age-related incidence in China showed an “S-shaped” rise, while that in India showed an “inverted U-shaped” rise. The overall trend of both was similar to the global level and was on the rise, but completely opposite to that in the United States. In China, the overall incidence of depressive disorders increases with age, and the incidence of depressive disorders is highest in the senior age group, mainly concentrated in the 85–89 age group. In addition, we can also observe a greater difference between the incidence rates of the high and low age groups as the years increase, with the most significant change in 2019, from 2153.63 per 100,000 in the 20–24 age group to 5799.83 per 100,000 in the 85–89 age group. In India, the overall trend is similar to that in China, except that the incidence of depressive disorders in India decreases with increasing age after 60–64 years, which is not obvious in 2014 and 2019. The global trend of depressive disorders incidence with age is almost the same as in India, except that the incidence is generally lower. In the United States, the overall incidence of depressive disorders declines with age, mainly in the 20–24 age group; In addition, the effect of time change on the incidence in the high and low age groups was not particularly significant. The exact values are indicated in Supplementary Table S3.

**Figure 4:**
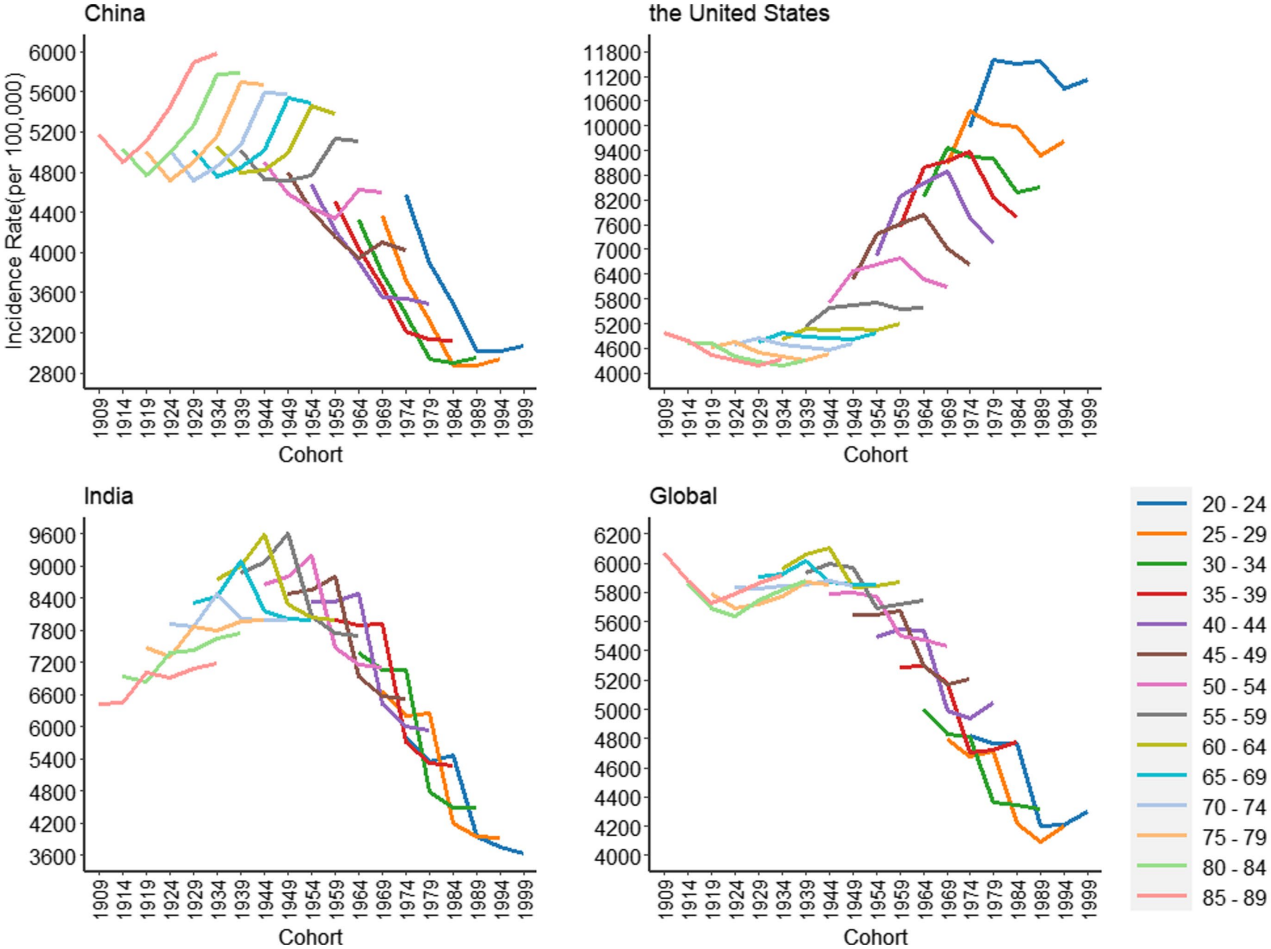
Cohort-based variation in age-standardized incidence rates for depressive disorders in China, the United States, India and the global, 1990 to 2019.

As is shown in Figure 4, the incidence of depressive disorders fluctuates significantly with birth cohort in China, the United States, India, and globally. The trend in China, India, and the world as a whole has been declining with increasing birth cohorts, and the opposite applies in the United States. In China, the incidence decreased with the increase of the birth cohort, and in the United States, the incidence increased with the increase of the birth cohort. In the same age group, the incidence of the 55–89 age group in China increased with the increase of the birth cohort, and the incidence of the 20–54 age group decreased with the increase of the birth cohort. In the United States, the overall trend has remained relatively stable. In addition, we found that the effects of age and cohort on the incidence of psychiatric disorders were interactional and could not be demonstrated independently. Therefore, we use the age-period-cohort model for a more accurate analysis. The exact values are shown in Supplementary Table S4.

### 3.5. The age, period, and cohort effects on depressive disorders incidence

#### 3.5.1. Age effects

**Figure 5:**
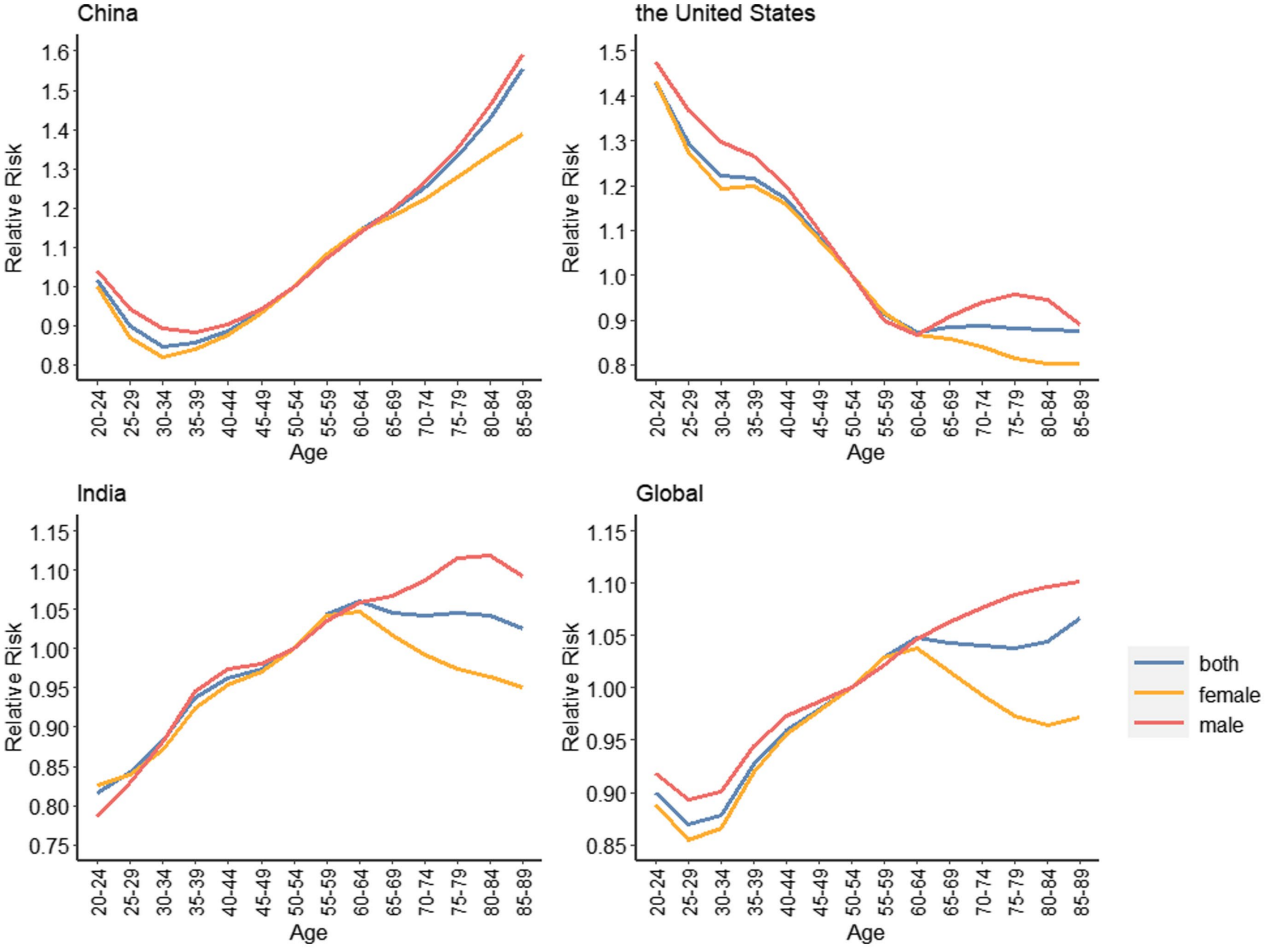
Age effects on depressive disorders incidence rate for China, the United States, India and the global, 1990–2019.

Figure 5 shows the risk ratio (RR) of depressive disorders incidence by age and sex in China, America, India and the world. Through analysis, it is found that the overall trend of China, India and the world is similar, which increases with age. To be more precise, China is rising in a U-shaped curve and India is rising in an approximately inverted U-shaped curve. But is opposite to that of the United States. In China, the age group of 85–89 has the highest risk of disease, with little gender difference. In India and the world, after the age group of 60–64 years old, the gender difference is more obvious. The risk of disease in women decreases, while that in men continues to increase; in the United States, the 20–24 age group is at the highest risk. After the age of 60–64, there is a gender difference. Women continue to decline, while men slightly increase.

#### 3.5.2. Period effects

**Figure 6:**
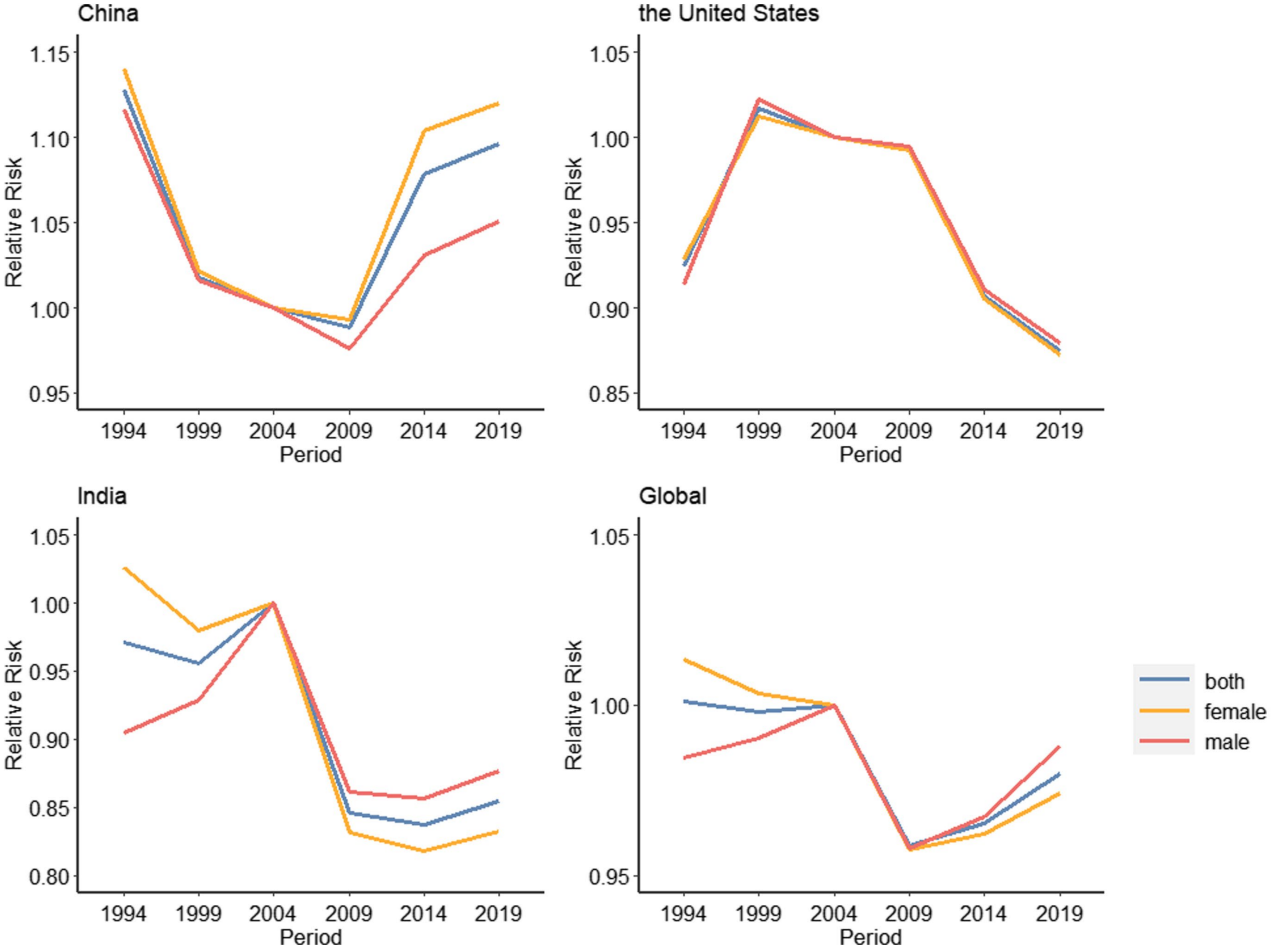
Period effects on depressive disorders incidence rate for China, the United States, India and the global, 1990–2019.

Figure 6 shows the period changes of depressive disorders risk in China, the United States, India and the world. The incidence risk in China decreased rapidly with the increase of the period before 1999, and then decreased slowly. Since 2009, it has build up over the increase of the period, and there is a gender difference. The increase of incidence risk of women is higher than that of men; the United States increased with the increase in the period before 1999, and then decreased slowly. Since 2009, it has declined sharply, with no significant difference between men and women; India is the same as the global tendency. Since 2004, it has declined rapidly with the increase of the period. In 2009, it rose slowly.

#### 3.5.3. Cohort effects

**Figure 7:**
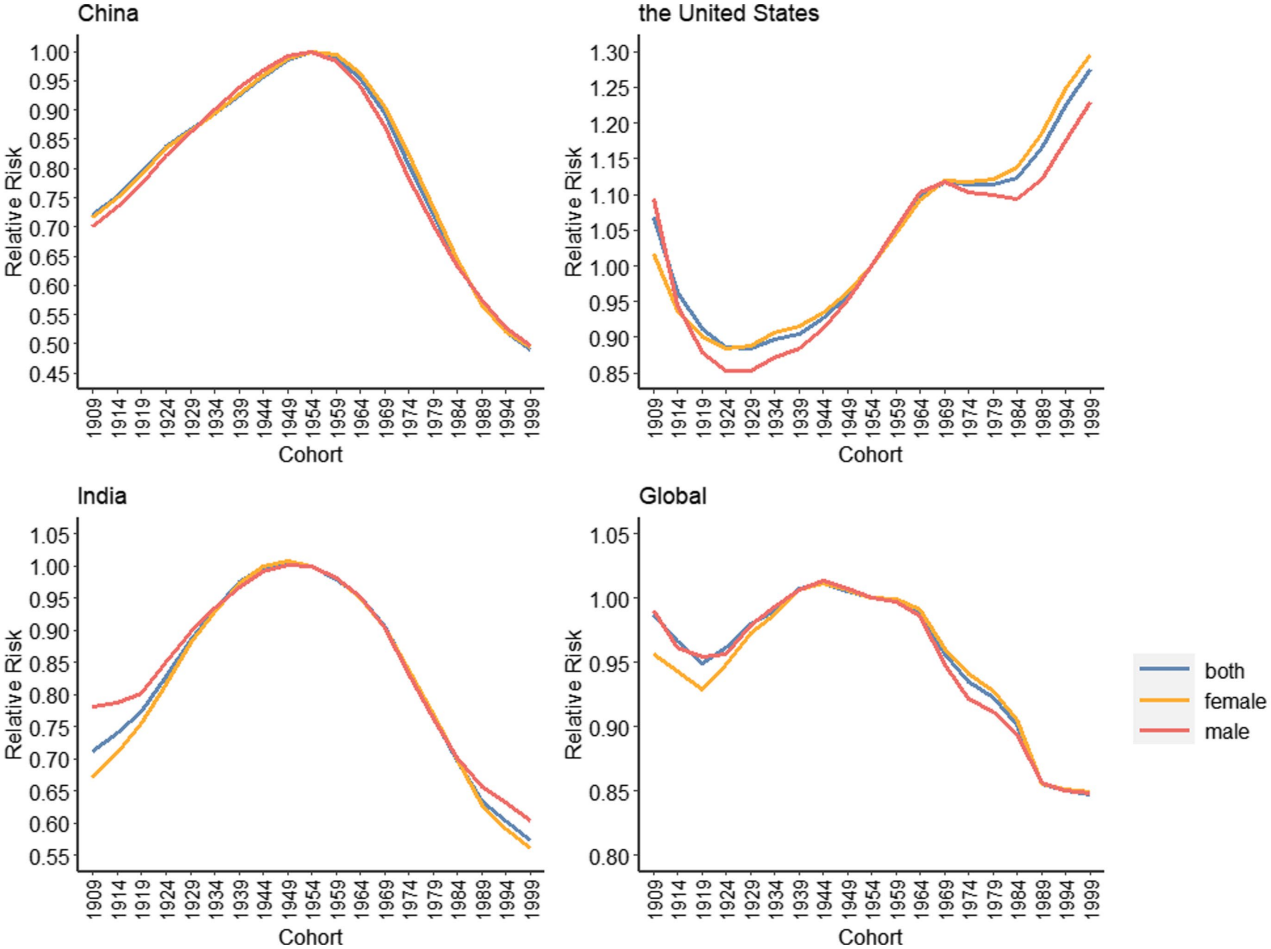
Cohort effects on depressive disorders incidence rate for China, the United States, India and the global, 1990–2019.

Figure 7 shows the RR trend of depressive disorders by cohort in China, the United States, India and the world. The trend of China and India is similar, showing an “inverted U” curve, and the mirror image of the United States. The birth cohort around 1954 holds the highest risk of disease. The risk of disease in the United States was the lowest around 1924, and then gradually increased. There is not any significant gender difference between China, America, India and the world. The exact values are shown in Supplementary Table S5.

## 4. Discussion

According to our findings, the ASIR of mental disorders declined slightly or stabilized in China, similar to global levels, but increased in the United States and decreased in India. Furthermore, women outnumber men in China, the United States, India, and around the world. This sex difference has been found and confirmed in previous studies (Phillips et al., 2009; Gu et al., 2013; Huang et al., 2019). This is due to the fact that a female’s life is divided into several stages, including menstruation, pregnancy, and menopause (Desai and Jann, 2000). Based on this, it is still necessary to analyze the trends in the incidence of mental disorders and explore the reasons and differences underlying these tendencies.

The study on depressive disorders found that from 1990 to 2019, the ASIR of depressive disorders in China decreased or stabilized slightly, similar to the global level, but the ASIR in the United States increased, while the ASIR of India decreased. Moreover, consistent with mental disorders, the morbidity of women is higher than that of men. This is in line with previous research results (Zhao et al., 2012; Faravelli et al., 2013). This may be because women are more likely to experience events that lead to depressive disorders, including sexism, physical and sexual abuse, the breakdown of close relationships, domestic violence, prenatal and postnatal pressure from family and parenting, and backward cultural norms (Patel et al., 2002; Keita, 2007). In addition, differences in stress tolerance levels between males and females also contribute to differences in depressive disorders rates between men and women (Piccinelli and Wilkinson, 2000). At the national level, the overall incidence of depressive disorders is the highest in the United States. Previous research has found that high-income North American and European countries have a higher burden of depressive disorders than Asian countries (Ferrari et al., 2013). This could be due to a lack of focus on mental illness in many developing countries, resulting in significant underreporting of such disorders, or a lack of high-quality epidemiological data (Bromet et al., 2011; Ferrari et al., 2013).

In order to analyze the reasons behind the trend of depressive disorders incidence rate, we could discuss the incidence rate changes from age, period and cohort perspective.

### 4.1. Age effects

In addition to other factors, there is a fairly obvious link between age and depressive disorders. Age has a significant impact on the incidence of depressive disorders in China, the United States, and India. The overall incidence of depressive disorders in China is increasing with age. In particular, the incidence is relatively high in the 20–24 age group, then begins to decline until the 30–34 age group, and then increases with age. The high incidence of the 20–24 age group may be related to Chinese peers’ competitiveness (Gao et al., 2012). People are going to college or just entering society at this point, separated from their parents and even protected by the school. They face social beatings and a variety of social pressures, including work pressure, economic pressure, and difficulties dealing with interpersonal relationships at work (Hasler, 2010). Furthermore, people at this age are still immature under immense pressure, so they are prone to indulge in the Internet, neglect social networking, and form an isolated personality (Yan et al., 2014). For various reasons, people in this age group are more likely to suffer from depressive disorders and have a higher incidence. People’s personalities mature as they age, and they become better at adjusting their emotions. The incidence gradually declined until the 30–34 age group. The main reason is that as they get older, their ability to learn new things and work begins to deteriorate. They face the additional burden of caring for both the elderly and children as they reach middle age, and their life pressure is multiplied (Lachman, 2002). The high incidence of depressive disorders in the elderly is caused by a variety of reasons. First of all, with the increase of age, people’s physical functions continue to decline, and they are more likely to suffer from various diseases, such as hypercortisolemia, hypertension and diabetes (Brown et al., 2004), as well as injuries and even physical disabilities. In addition, muscle strength tends to weaken and hearing loss occurs with age, and previous studies have linked muscle weakness and age-related hearing loss to depressive disorders (Chen et al., 2020; Feng et al., 2022). Besides, some drugs used to treat diseases of the elderly may have side effects and increase the risk of depressive disorders (Beers and Passman, 1990; You et al., 2013). Aside from these physical factors, some social factors, such as empty nest elderly (Zhai et al., 2015; Wang et al., 2017), lower educational attainment, disconnection from modern society (Du et al., 2022) and loss of spouses (Xu et al., 2020) are also major contributors to the high incidence of depressive disorders among the elderly (Feng et al., 2019). Additionally, the Chinese people and their government have traditionally anticipated that the family should provide and care for the elderly, partly rooted in filial piety (Cheng et al., 2013; Chou et al., 2015; Fung and Jiang, 2016), making social security in China largely inadequate. These elements collectively enhance the danger for older persons.

The incidence declines with age in the United States, which exhibits the reverse tendency. In the United States, the incidence of the elderly is low, probably because the pension system is more perfect and the pension model is more advanced. In 1935, the United States Congress passed the Social Security Act (Quadagno, 1984), the Older Americans Act in 1965 (Binstock, 1991), the Social Security Act in 1974, and the Medicare Act, the Health Care Plan, etc.… In the United States, the “combination of medical care and pension “has been applied for a long time, and there are also community elderly care, wise elderly care, and so on. A good aged care system keeps the elderly at ease and reduces their risk of developing depressive disorders. As for young people, we all know that the United States has numerous colleges and universities, and the competition is very severe, which invisibly increases their pressure. According to the US census data, more than 30% of young people in the United States are uninsured (DeNavas-Walt, 2010), that is, they do not have adequate social security. Various reasons lead to the high incidence of adolescent depressive disorders.

The risk of disease in India increases with age. Previous studies have found that compared with the 18–59 age group, the elderly are 1.35 times more likely to suffer from depressive disorders (Sinha et al., 2021). The WHO’s 2015 estimates for different age groups (Organization WH, 2017) and the study of SAGE countries, including India (Brinda et al., 2016), also found the same results. Additionally, the incidence of high age group in India is higher than that in other countries, which is related to the food safety problem in India. Many previous studies have demonstrated a connection between food safety and mental health, especially geriatric depressive disorders (Klesges et al., 2001; German et al., 2011).

### 4.2. Period effects

Regarding the period’s effect on the incidence of depressive disorders, we can see that it has a significant effect in China, America, and India. In China, RR has been falling from 1994 to 2009. From 1994 to 1999, the reduction was quick, but was gradual from 1999 to 2009. From 2009 to 2019, RR showed an upward trend, rising rapidly before 2014, and then slowing down. In the United States, from 1994 to 1999, there was an upward trend, and from 1999 to 2019, there was a downward trend. The decline was swift from 2009 to 2014, and the RR in 2019 was far lower than that in 1994. India, similar to the global, declined rapidly from 2004 to 2009 and then stabilized.

Specifically, the decline in the morbidity from 1994 to 2009 was mostly because of the rapid development of China’s society, the significant improvement in economic level and people’s education level in recent years (Qin et al., 2018). In addition, the Ministry of Finance supported the mental health reform program in 2004, following the approval of the Proposal on Further Strengthening Mental Health Work (Liu et al., 2011). Later, the “Central Subsidized Local Health Funds Management and Treatment Project for Serious Mental Diseases” further consolidated the achievements of mental health reform in 2006 (Liang et al., 2018). The formulation of these public health policies is what caused the drop in the incidence rate of depression throughout this time. While the slowdown in the incidence rate in 1999–2009 was due to severe acute respiratory syndrome (SARS), the impact of the Wenchuan earthquake and the economic crisis. The burst of SARS in 2003 led to the whole society to fall into extreme panic. The medical staff in charge of prevention and control work were caught up in intense work, and people who were isolated were more likely to suffer from depressive disorders due to fear and loneliness (Cheng et al., 2004; Liu et al., 2012). The 2008 Wenchuan earthquake caused huge contretemps. Many people lost their relatives in grief. Also, the earthquake caused serious economic losses, which in turn affected the risk of depressive disorders (Kun et al., 2010). Previous studies have found that earthquake has an “anniversary effect” on depression, that is, depression symptoms tend to be more severe 12 and 24 months after the earthquake (Ye et al., 2014; Zhou et al., 2016) and correspondingly reported higher incidence. The financial crisis in 2008 made countless people confront the crisis of joblessness and increased the pressure on their lives (Lee et al., 2010; Wang et al., 2010). The H1N1 outbreak in 2009 caused panic and even depressive disorders because people did not know about the virus (Wang and Wang, 2021). The 2010 Southwest China earthquake occurred in a densely populated area, and the houses were easily destroyed, which exacerbated the damage and casualties and brought a huge psychological impact to residents (Zhang et al., 2012). In recent years, with the continuous expansion of higher education, there is a serious mismatch between the supply and demand of labor force, and more and more young people say that it is difficult to find a job and the pressure of employment is great (Hua and Ma, 2022). These natural and social factors have contributed to the increasing risk of depressive disorders since 2009.

The above tendency in the United States is mainly as a result of the following factors: First, the increase in the incidence rate from 1994 to 1999 was mainly because of some events in these years, the large earthquake in 1994, which affected Los Angeles, Santa Clarita, Fillmore, and many other cities have had a severe impact (Bourque et al., 1994); A terrorist attack in 1995 - the Oklahoma City bombing caused a large number of casualties and extreme social panic (Krug et al., 1996); The Boeing 747 explosion in 1996 and the Florida riots and more. These events all contribute to a certain degree of increased incidence of depressive disorders. After 1999, the number of wars in the United States decreased, and peace gradually recovered from the war. People have fled from panic, and due to the previous major earthquakes, riots, etc., the United States’ disaster mental health service system has been improving day by day (Jacobs, 1995). In addition, community mental health services are becoming ever more effective (Drake and Latimer, 2012), so the incidence rate has begun to show a downward trend.

In India, the risk of depressive disorders declined sharply around 2004. According to previous studies, the rural poverty rate in India decreased around 2004 (Lanjouw and Murgai, 2009; Naraparaju and Chandrasekhar, 2021). In addition, poverty has been shown to be strongly associated with depressive. Therefore, the reduced risk of depressive disorders in India may be associated with poverty reduction (Lino et al., 2013; Baiyewu et al., 2015; Cheung and Chou, 2019; Lee and Chou, 2019).

### 4.3. Cohort effects

For the cohort effect, China and the United States showed opposite patterns of change, similar to India and the global average.

In China, the incidence of depressive disorders was on the rise before 1954 and the decline after 1954. The incidence in 1999 was much lower than that in 1909. The higher incidence of depressive disorders in the pre-1954 birth cohort is mainly due to the fact that they were born during the 1911 Revolution, the 1931–1945 Anti-Japanese War and the 1927–1949 Civil War, when the upheavals of these periods produced great worry and anxiety and caused great pressure which increases the risk of depressive disorders. The WHO estimates that about 10 percent of the population will undergo serious mental health problems after the war, such as depressive disorders (Saraceno, 2002). Previous studies have shown that war significantly increases the incidence and prevalence of depressive disorders, and that war exacerbates this trend by causing various physical injuries, such as disability (Sareen et al., 2007). Those who were born between 1949 and 1954, a post-war period with some lingering impacts from past wars, were at the greatest risk. In addition, during this period, new China had just been liberated, and people still experienced some historical events in their childhood, adolescence and youth, such as the Great Leap Forward, famine and the Cultural Revolution. In their middle and old age, they suffered from the SARS epidemic and Wenchuan earthquake. These are all risk factors for depressive disorders in this birth cohort (Lee, 1999; Lester, 2005). The risk for those who were born after 1954 continuously decreased, reaching its lowest point in 1999. This is mainly due to the founding of New China. People got rid of the suffering of wars and turmoil, and they were freed from displacement. The social economy grew rapidly, and the level of education continued to improve. Medical and health services have been uninterruptedly strengthened. Living circumstances have consistently improved, and people are happier and healthier than before.

In the United States, RR showed a downward trend from 1909 to 1924, and an overall upward trend from 1924 to 1999. Among them, it stabilized from 1969 to 1974. The overall trend is upward. The decline in morbidity in the 1909–1924 birth cohort in the United States may be due to the emergence of the modern mental health movement in 1908, which made people realize the significance of mental health. Incidence rates in subsequent birth cohorts increased year by year mainly due to the Great Depressive disorders of the 1930s, World War II in the 1940s, etc. The 1969–1974 leveling off may be linked to the Community Mental Health Act enacted in 1968 (Levine, 1981). Incidence rates in subsequent birth cohorts continued to rise, which was highly relevant to the rapid development of the U.S. economy and technology, which resulted in a surge in people’s stress, increased employment pressure, and a susceptibility to depressive disorders.

Before 1947, India had been subjected to British colonial rule. During this period, the British government’s oppression of the Indian people increased the risk of depressive disorders (Sundar, 2011). In addition, the famine in India in 1946 caused the Indian people to be hungry, which also increased the risk of depressive disorders among people born during this period (Passmore, 1951). Since 1970, India has taken relevant measures to solve mental health problems, including funding for mental health programs and encouraging governments at all levels to implement mental health laws (Sharma, 2014; Gupta and Sagar, 2022). Furthermore, India launched a national mental health project in 1982 (Duffy and Kelly, 2019). This series of measures reduced the risk of the disease during this period and in the birth cohort.

In addition, it is worth mentioning that this study has certain limitations. First, because this article is built on the data of GBD 2019, there is still some deviation in the completeness and accuracy of the incidence data of mental disorders and depressive disorders that we use, making the results less accurate. Second, we do not possess detailed incidence data for each country and region, and cannot analyze the spatial distribution.

## 5. Conclusion

In conclusion, from 1990 to 2019, the ASIR of depressive disorders in China and India decreased slightly or remained the same, while that in the United States showed an upward trend, and the incidence of women in the three countries was higher than that of men. As far as the age effect is concerned, China and India are on the rise, while the United States is on the decline. As far as the cyclical effect is concerned, China has shown an upward tendency in recent years, while the United States and India have shown a downward trend. In terms of queue effect, China and India showed a downward tendency as a whole, while the United States showed an upward trend. Both period and cohort effects are related to national policies and whether there is a war. We should take the development of depressive disorders into account, and regulate coping strategies on the basis of the cohort effect of age to effectively reduce the occurrence of such diseases.

## Data availability statement

The datasets presented in this study can be found in online repositories. The names of the repository/repositories and accession number(s) can be found below: the Institute for Health Metrics and Evaluation, http://www.healthdata.org/.

## Author contributions

CY supervised the study. CY and SW designed the study. SW, TL, RL, TW, LH, and JS collected the data. SW and TL organized the data, analyzed the data, and interpreted the results. SW wrote the first draft. CY, SW, TL, RL, TW, LH, and JS reviewed and edited the manuscript. All authors contributed to the article and approved the submitted version.

## Funding

This work was funded by the National Natural Science Foundation of China (Grant Nos. 82173626 and 81773552) and Health commission of Hubei Province scientific research project (Grant No. WJ2019H304).

## Conflict of interest

The authors declare that the research was undertaken in the absence of any commercial or financial relationships that could be construed as a potential conflict of interest.

## Publisher’s note

All claims expressed in this article are solely those of the authors and do not necessarily represent those of their affiliated organizations, or those of the publisher, the editors and the reviewers. Any product that may be evaluated in this article, or claim that may be made by its manufacturer, is not guaranteed or endorsed by the publisher.
