# Supplementary material for: Long-term trends in the incidence of depressive disorders in China, the United States, India and globally: A comparative study from 1990 to 2019
Source: Front Psychol. 2023 Jan 17;13:1066706. doi: 10.3389/fpsyg.2022.1066706 (PMC9888314; doi:10.3389/fpsyg.2022.1066706)
Supplement: Supplementary file 1 [file Table_1.DOCX]

Supplementary Material

# Supplementary Tables

**Table S1** Age-standardized incidence rates (ASIR) for mental disorders in males and females in China, the United States, India and the global, 1990 to 2019.

| Year | China  (Coef, 95% CI) | | | the United States  (Coef, 95% CI) | | | India  (Coef, 95% CI) | | | Global  (Coef, 95% CI) | | |
| --- | --- | --- | --- | --- | --- | --- | --- | --- | --- | --- | --- | --- |
|  | both | males | females | both | males | females | both | males | females | both | males | females |
| 1990 | 3604.93 (3260.70,3954.82) | 2833.22 (2583.55,3097.12) | 4409.83 (3951.49,4872.18) | 5253.37 (4748.98,5780.91) | 4247.52 (3860.58,4649.38) | 6221.41 (5587.39,6888.34) | 5393.32 (4790.47,6052.84) | 4485.81 (4014.02,5002.99) | 6383.52 (5616.92,7227.51) | 4720.70 (4246.89,5212.86) | 3806.57 (3438.58,4181.52) | 5619.83 (5030.62,6221.36) |
| 1991 | 3694.70 (3351.35,4054.78) | 2962.46 (2697.72,3233.23) | 4457.31 (4004.29,4921.11) | 5418.72 (4894.95,5979.99) | 4358.92 (3954.13,4781.81) | 6441.43 (5783.08,7139.71) | 5615.28 (4985.80,6311.75) | 4603.65 (4115.94,5142.72) | 6717.63 (5915.48,7593.77) | 4773.49 (4297.40,5271.74) | 3854.79 (3475.33,4238.70) | 5678.05 (5097.54,6286.72) |
| 1992 | 3763.15 (3413.88,4126.54) | 3066.93 (2789.80,3351.39) | 4487.03 (4038.81,4948.92) | 5585.93 (5040.76,6173.72) | 4472.40 (4043.07,4917.72) | 6663.48 (5984.17,7408.17) | 5808.95 (5160.36,6533.80) | 4708.71 (4210.25,5276.18) | 7005.62 (6178.09,7921.05) | 4818.70 (4340.59,5329.14) | 3896.32 (3521.03,4281.03) | 5727.70 (5137.61,6351.83) |
| 1993 | 3809.40 (3451.05,4170.08) | 3144.78 (2859.35,3439.54) | 4499.00 (4063.79,4955.39) | 5749.21 (5177.45,6351.26) | 4584.62 (4144.19,5038.58) | 6879.36 (6173.48,7674.85) | 5963.03 (5304.08,6710.68) | 4795.26 (4288.52,5375.88) | 7230.01 (6366.33,8173.52) | 4854.25 (4377.01,5366.71) | 3929.66 (3560.33,4317.54) | 5765.97 (5176.81,6392.30) |
| 1994 | 3832.10 (3477.49,4193.63) | 3193.89 (2906.63,3487.95) | 4492.93 (4064.98,4940.54) | 5902.85 (5312.18,6523.26) | 4692.23 (4245.84,5156.75) | 7080.79 (6348.47,7919.09) | 6066.10 (5393.06,6830.08) | 4857.42 (4336.56,5452.52) | 7373.19 (6490.38,8331.32) | 4878.30 (4398.91,5393.41) | 3953.34 (3580.03,4349.19) | 5790.57 (5203.65,6418.31) |
| 1995 | 3830.03 (3480.19,4186.00) | 3211.69 (2929.28,3505.35) | 4468.79 (4049.67,4906.04) | 6040.92 (5429.99,6704.96) | 4791.83 (4325.58,5275.33) | 7259.47 (6500.64,8125.20) | 6106.43 (5417.27,6883.29) | 4889.22 (4357.31,5491.70) | 7417.72 (6537.04,8380.04) | 4888.64 (4410.49,5400.71) | 3965.90 (3592.43,4361.17) | 5798.54 (5213.32,6427.24) |
| 1996 | 3790.24 (3448.70,4137.55) | 3186.93 (2903.86,3472.88) | 4411.95 (4006.09,4836.56) | 6216.42 (5593.95,6889.64) | 4920.02 (4440.98,5426.60) | 7485.34 (6714.97,8362.84) | 6088.39 (5409.03,6858.02) | 4891.75 (4358.67,5487.70) | 7372.17 (6494.66,8334.06) | 4889.01 (4413.00,5397.85) | 3969.37 (3595.46,4360.63) | 5795.57 (5214.28,6419.90) |
| 1997 | 3714.71 (3387.45,4046.22) | 3124.22 (2852.82,3400.53) | 4321.81 (3927.70,4727.28) | 6450.45 (5818.24,7144.10) | 5089.71 (4600.88,5617.34) | 7788.21 (6995.46,8687.55) | 6038.58 (5355.73,6797.23) | 4878.37 (4355.62,5467.26) | 7278.00 (6410.82,8217.43) | 4884.86 (4412.46,5389.26) | 3967.52 (3600.15,4352.78) | 5788.98 (5210.05,6410.16) |
| 1998 | 3624.92 (3309.52,3939.50) | 3045.18 (2786.26,3308.58) | 4219.44 (3842.82,4599.54) | 6689.83 (6051.49,7399.47) | 5262.01 (4756.80,5810.22) | 8099.65 (7290.99,9003.06) | 5978.46 (5308.67,6736.34) | 4861.35 (4341.34,5449.19) | 7166.98 (6313.69,8099.96) | 4877.93 (4409.65,5377.35) | 3962.63 (3598.12,4341.32) | 5779.87 (5208.89,6397.12) |
| 1999 | 3542.72 (3242.36,3842.68) | 2972.51 (2723.44,3228.58) | 4125.90 (3760.01,4488.93) | 6881.72 (6234.61,7603.03) | 5398.23 (4874.71,5958.35) | 8351.52 (7526.19,9278.84) | 5929.63 (5255.26,6680.05) | 4853.05 (4322.59,5437.01) | 7070.82 (6231.15,7998.82) | 4870.55 (4405.98,5364.50) | 3957.46 (3595.26,4332.18) | 5770.10 (5200.38,6380.00) |
| 2000 | 3489.44 (3201.22,3777.63) | 2927.65 (2684.38,3174.71) | 4062.08 (3703.60,4420.57) | 6973.24 (6318.82,7696.76) | 5459.76 (4937.39,6026.93) | 8475.88 (7643.88,9412.78) | 5913.88 (5238.83,6660.61) | 4865.89 (4335.36,5453.83) | 7020.91 (6179.65,7935.37) | 4864.73 (4402.48,5354.84) | 3954.32 (3592.40,4329.44) | 5761.32 (5192.25,6363.96) |
| 2001 | 3462.17 (3179.66,3745.59) | 2909.30 (2667.96,3156.11) | 4023.34 (3668.12,4376.91) | 6980.41 (6328.79,7707.49) | 5456.33 (4947.50,6021.20) | 8495.44 (7658.88,9439.28) | 5942.94 (5265.96,6696.41) | 4911.89 (4375.61,5505.74) | 7028.42 (6195.85,7961.52) | 4863.21 (4400.90,5352.44) | 3956.11 (3592.02,4331.78) | 5756.21 (5190.93,6358.61) |
| 2002 | 3441.67 (3164.94,3723.54) | 2899.28 (2660.95,3141.84) | 3989.79 (3636.90,4340.06) | 6963.47 (6313.10,7686.91) | 5431.68 (4918.71,5992.68) | 8487.89 (7645.40,9420.91) | 5999.97 (5317.34,6764.60) | 4981.62 (4438.66,5581.11) | 7068.87 (6231.63,8016.73) | 4865.04 (4402.89,5354.66) | 3961.67 (3594.40,4340.01) | 5754.17 (5191.53,6358.13) |
| 2003 | 3424.44 (3149.74,3705.23) | 2892.98 (2652.17,3131.88) | 3959.17 (3615.86,4305.97) | 6935.81 (6284.61,7654.28) | 5398.08 (4886.76,5950.64) | 8467.68 (7631.49,9394.39) | 6064.28 (5375.11,6836.17) | 5055.62 (4500.89,5661.63) | 7120.14 (6274.21,8070.14) | 4867.73 (4406.36,5358.31) | 3968.12 (3597.42,4348.91) | 5752.98 (5191.35,6360.01) |
| 2004 | 3407.41 (3132.60,3685.65) | 2886.66 (2642.38,3130.22) | 3929.41 (3590.97,4272.92) | 6910.90 (6266.20,7624.56) | 5367.83 (4862.73,5908.98) | 8449.29 (7616.42,9380.00) | 6115.04 (5418.68,6891.75) | 5114.25 (4551.40,5733.02) | 7160.12 (6314.44,8121.42) | 4868.95 (4408.10,5360.63) | 3972.84 (3599.59,4356.05) | 5750.58 (5192.49,6359.91) |
| 2005 | 3387.52 (3114.33,3661.62) | 2875.96 (2629.74,3119.16) | 3898.59 (3563.97,4235.42) | 6902.24 (6255.17,7607.38) | 5353.38 (4844.59,5887.83) | 8447.30 (7615.49,9370.16) | 6131.30 (5431.35,6919.59) | 5137.85 (4567.67,5764.37) | 7166.57 (6326.60,8135.20) | 4865.81 (4406.47,5361.20) | 3972.76 (3598.32,4357.57) | 5744.17 (5181.94,6356.39) |
| 2006 | 3355.48 (3082.48,3626.93) | 2853.83 (2612.07,3095.01) | 3855.13 (3527.52,4181.89) | 6917.82 (6259.52,7622.04) | 5359.00 (4852.95,5896.65) | 8473.51 (7628.23,9372.65) | 6003.49 (5334.03,6760.05) | 5048.96 (4500.85,5640.19) | 6996.16 (6186.53,7906.30) | 4835.07 (4385.10,5322.97) | 3951.17 (3585.53,4326.13) | 5704.10 (5152.16,6305.88) |
| 2007 | 3311.66 (3040.69,3580.99) | 2821.82 (2582.52,3059.55) | 3798.18 (3478.18,4112.81) | 6948.66 (6283.92,7648.15) | 5375.42 (4873.66,5910.40) | 8519.35 (7664.99,9417.09) | 5709.84 (5098.47,6396.13) | 4837.54 (4324.24,5388.36) | 6614.54 (5886.45,7421.93) | 4770.26 (4337.30,5243.20) | 3904.41 (3543.33,4270.67) | 5621.12 (5086.79,6201.14) |
| 2008 | 3267.25 (3004.08,3533.83) | 2788.20 (2553.23,3025.19) | 3741.70 (3428.93,4044.15) | 6982.80 (6316.04,7682.46) | 5394.89 (4894.55,5934.25) | 8568.69 (7706.07,9471.75) | 5360.49 (4803.72,5954.89) | 4584.92 (4116.76,5091.56) | 6162.05 (5511.32,6860.87) | 4694.84 (4273.82,5151.64) | 3849.97 (3498.44,4210.79) | 5524.50 (5008.09,6077.12) |
| 2009 | 3233.02 (2972.68,3495.10) | 2760.91 (2530.64,2993.92) | 3699.52 (3392.19,4002.44) | 7008.19 (6338.77,7705.30) | 5409.48 (4907.90,5958.64) | 8605.18 (7752.71,9516.55) | 5064.78 (4562.93,5612.87) | 4371.86 (3940.09,4839.59) | 5777.98 (5185.35,6409.61) | 4631.83 (4217.69,5076.04) | 3804.83 (3462.50,4160.64) | 5443.32 (4933.59,5973.96) |
| 2010 | 3219.89 (2962.68,3479.66) | 2747.81 (2519.13,2980.72) | 3685.60 (3380.89,3987.21) | 7012.67 (6341.95,7719.88) | 5411.36 (4906.48,5969.58) | 8612.38 (7742.61,9527.65) | 4931.36 (4449.78,5457.06) | 4278.73 (3850.92,4736.89) | 5600.71 (5032.56,6209.81) | 4603.88 (4192.83,5043.68) | 3785.71 (3446.63,4143.63) | 5406.29 (4899.46,5930.28) |
| 2011 | 3230.48 (2971.80,3492.97) | 2747.45 (2517.96,2980.17) | 3706.62 (3398.19,4016.03) | 6958.34 (6304.51,7659.42) | 5372.47 (4870.02,5902.91) | 8542.52 (7689.42,9459.44) | 4921.06 (4442.32,5439.34) | 4277.79 (3861.92,4730.92) | 5579.18 (5006.81,6173.47) | 4603.62 (4188.39,5049.13) | 3788.04 (3448.78,4142.06) | 5403.42 (4895.19,5932.89) |
| 2012 | 3254.59 (2990.85,3521.70) | 2751.82 (2521.94,2987.10) | 3750.15 (3433.01,4072.92) | 6841.84 (6191.87,7532.93) | 5289.03 (4800.52,5814.87) | 8392.77 (7568.07,9302.12) | 4920.03 (4442.89,5435.68) | 4285.12 (3866.38,4736.74) | 5568.14 (5004.21,6170.83) | 4606.98 (4187.58,5059.31) | 3794.12 (3450.12,4154.50) | 5404.17 (4880.68,5942.76) |
| 2013 | 3283.62 (3012.12,3559.34) | 2758.51 (2526.22,2998.87) | 3801.25 (3475.28,4138.18) | 6702.81 (6065.94,7380.62) | 5189.62 (4709.07,5708.71) | 8213.90 (7410.16,9101.12) | 4924.20 (4443.07,5440.86) | 4296.47 (3875.33,4753.37) | 5563.66 (4996.52,6175.71) | 4612.98 (4184.85,5073.26) | 3802.56 (3457.78,4164.41) | 5407.83 (4877.06,5958.87) |
| 2014 | 3309.39 (3032.74,3591.80) | 2766.08 (2530.35,3010.10) | 3845.15 (3504.73,4203.23) | 6580.82 (5954.98,7244.23) | 5102.73 (4629.58,5612.24) | 8056.62 (7263.13,8933.85) | 4929.24 (4442.23,5448.29) | 4307.48 (3888.41,4760.51) | 5561.55 (4994.00,6177.42) | 4620.57 (4185.06,5089.39) | 3812.06 (3465.35,4172.85) | 5413.60 (4879.43,5982.10) |
| 2015 | 3323.00 (3042.37,3612.70) | 2771.44 (2534.75,3012.95) | 3866.84 (3527.07,4227.36) | 6515.47 (5887.27,7177.62) | 5056.81 (4586.72,5560.40) | 7971.73 (7176.41,8843.54) | 4930.58 (4441.43,5466.75) | 4313.44 (3886.93,4760.36) | 5557.50 (4981.73,6172.34) | 4628.30 (4186.32,5105.29) | 3821.00 (3471.19,4189.38) | 5420.30 (4881.48,5989.01) |
| 2016 | 3327.95 (3044.50,3614.18) | 2779.24 (2543.11,3024.60) | 3868.07 (3526.60,4221.41) | 6505.78 (5888.29,7179.70) | 5055.02 (4575.88,5551.11) | 7953.93 (7193.49,8828.52) | 4930.36 (4442.75,5451.09) | 4317.00 (3891.84,4752.56) | 5553.02 (4981.83,6158.62) | 4635.30 (4191.64,5113.63) | 3829.53 (3481.51,4193.98) | 5426.02 (4885.72,5999.56) |
| 2017 | 3331.83 (3049.46,3615.46) | 2787.40 (2548.82,3036.93) | 3866.90 (3520.92,4212.03) | 6508.15 (5906.43,7186.25) | 5060.55 (4589.24,5558.56) | 7952.86 (7206.85,8841.95) | 4929.17 (4439.89,5446.73) | 4319.19 (3889.52,4748.18) | 5548.13 (4982.78,6152.05) | 4642.85 (4194.58,5120.12) | 3838.63 (3487.81,4207.72) | 5432.33 (4883.21,6012.01) |
| 2018 | 3324.57 (3042.45,3620.51) | 2783.59 (2545.01,3028.45) | 3856.69 (3517.77,4222.23) | 6526.36 (5908.77,7199.85) | 5076.77 (4595.48,5585.91) | 7972.80 (7184.52,8851.73) | 4916.80 (4438.27,5442.78) | 4314.44 (3896.48,4764.31) | 5527.73 (4959.42,6167.17) | 4651.57 (4196.78,5137.14) | 3849.56 (3484.55,4237.63) | 5439.26 (4877.84,6032.58) |
| 2019 | 3305.02 (3020.64,3612.66) | 2769.37 (2527.47,3010.79) | 3833.50 (3484.80,4209.92) | 6568.54 (5907.01,7245.67) | 5114.86 (4628.95,5629.52) | 8018.81 (7179.71,8917.51) | 4893.47 (4389.66,5442.25) | 4304.09 (3872.34,4754.24) | 5490.85 (4900.49,6150.38) | 4660.91 (4189.44,5161.64) | 3862.83 (3483.58,4251.39) | 5445.28 (4869.79,6051.33) |

**Table S2** Age-standardized incidence rates (ASIR) for depressive disorders in males and females in China, the United States, India and the global, 1990 to 2019.

| Year | China  (Coef, 95% CI) | | | the United States  (Coef, 95% CI) | | | India  (Coef, 95% CI) | | | Global  (Coef, 95% CI) | | |
| --- | --- | --- | --- | --- | --- | --- | --- | --- | --- | --- | --- | --- |
|  | both | males | females | both | males | females | both | males | females | both | males | females |
| 1990 | 2647.72 (2335.50,2991.38) | 1831.44 (1616.72,2062.27) | 3500.38 (3065.29,3955.94) | 3849.98 (3401.99,4356.91) | 2776.83 (2454.34,3156.20) | 4891.45 (4313.84,5532.18) | 4520.76 (3944.13,5161.57) | 3569.28 (3123.40,4064.89) | 5558.26 (4830.18,6359.53) | 3681.24 (3239.27,4150.12) | 2735.94 (2415.75,3086.22) | 4613.68 (4064.28,5203.44) |
| 1991 | 2730.69 (2413.14,3073.77) | 1950.88 (1721.13,2190.83) | 3544.79 (3123.94,3982.96) | 4008.23 (3537.97,4551.57) | 2883.97 (2550.15,3273.86) | 5101.19 (4494.19,5796.40) | 4745.09 (4143.95,5415.82) | 3686.77 (3235.09,4203.18) | 5897.83 (5139.78,6764.94) | 3733.30 (3288.52,4204.27) | 2782.60 (2456.62,3134.89) | 4672.14 (4123.92,5267.71) |
| 1992 | 2792.78 (2474.54,3132.93) | 2046.59 (1806.64,2296.17) | 3571.27 (3165.40,4001.23) | 4167.62 (3681.47,4745.31) | 2993.28 (2647.85,3415.86) | 5311.46 (4678.34,6052.29) | 4941.44 (4318.79,5643.30) | 3792.24 (3323.57,4329.95) | 6191.13 (5389.12,7096.69) | 3777.77 (3333.11,4254.79) | 2822.83 (2493.33,3179.48) | 4721.73 (4176.15,5316.25) |
| 1993 | 2833.47 (2511.33,3175.61) | 2117.21 (1875.23,2378.04) | 3579.97 (3180.73,4010.32) | 4322.87 (3815.09,4928.82) | 3101.53 (2748.01,3541.48) | 5514.96 (4855.26,6302.47) | 5098.36 (4456.59,5831.89) | 3879.90 (3395.94,4444.79) | 6420.47 (5577.06,7361.22) | 3812.64 (3369.47,4293.20) | 2855.22 (2526.77,3214.34) | 4759.75 (4207.27,5361.92) |
| 1994 | 2851.81 (2533.86,3189.66) | 2161.27 (1915.17,2428.17) | 3570.80 (3178.27,3996.41) | 4468.81 (3948.88,5112.77) | 3205.50 (2832.40,3670.00) | 5704.37 (5024.36,6532.79) | 5204.30 (4544.86,5963.43) | 3943.75 (3443.12,4523.03) | 6567.99 (5714.64,7522.58) | 3836.29 (3399.99,4314.32) | 2878.50 (2552.44,3242.89) | 4784.09 (4237.14,5385.41) |
| 1995 | 2847.40 (2532.89,3179.13) | 2177.38 (1932.92,2435.40) | 3544.05 (3153.95,3965.34) | 4600.02 (4060.17,5272.55) | 3301.91 (2926.55,3783.38) | 5872.35 (5162.45,6716.25) | 5247.33 (4568.71,6018.11) | 3977.74 (3468.38,4565.44) | 6616.00 (5746.81,7598.14) | 3846.74 (3417.90,4331.39) | 2891.53 (2562.35,3264.10) | 4791.95 (4244.32,5396.07) |
| 1996 | 2803.99 (2497.67,3126.96) | 2152.40 (1913.63,2408.19) | 3480.42 (3103.04,3881.27) | 4754.12 (4195.16,5444.92) | 3415.22 (3028.24,3908.51) | 6070.07 (5334.59,6924.19) | 5235.08 (4562.46,5994.23) | 3987.52 (3480.19,4570.08) | 6574.87 (5709.59,7550.57) | 3846.56 (3416.93,4331.06) | 2896.11 (2567.65,3267.67) | 4786.87 (4240.90,5387.07) |
| 1997 | 2721.75 (2428.82,3030.58) | 2089.16 (1860.42,2332.56) | 3377.48 (3015.12,3764.46) | 4946.18 (4373.20,5642.73) | 3553.71 (3149.93,4065.27) | 6319.86 (5567.84,7190.64) | 5195.27 (4538.52,5938.70) | 3987.93 (3483.99,4575.32) | 6486.87 (5635.94,7434.74) | 3840.61 (3415.75,4318.14) | 2895.68 (2570.03,3264.25) | 4775.33 (4240.71,5370.58) |
| 1998 | 2624.87 (2350.31,2921.56) | 2010.26 (1795.86,2237.29) | 3260.84 (2922.41,3631.69) | 5137.60 (4550.46,5841.14) | 3689.84 (3266.81,4221.36) | 6571.14 (5802.28,7457.27) | 5146.06 (4486.52,5879.90) | 3986.48 (3481.27,4573.73) | 6382.02 (5545.10,7301.18) | 3831.57 (3411.54,4301.10) | 2892.43 (2571.06,3255.92) | 4760.41 (4233.29,5349.37) |
| 1999 | 2537.15 (2277.79,2820.20) | 1938.17 (1734.17,2150.77) | 3155.82 (2835.41,3511.20) | 5290.03 (4683.95,6008.21) | 3796.21 (3352.90,4339.46) | 6773.59 (5991.86,7669.11) | 5105.76 (4450.47,5831.17) | 3990.75 (3487.69,4576.97) | 6290.33 (5474.28,7193.42) | 3822.58 (3405.87,4285.49) | 2888.89 (2571.20,3248.33) | 4745.85 (4222.57,5328.90) |
| 2000 | 2482.65 (2230.02,2756.44) | 1895.35 (1699.63,2098.42) | 3087.88 (2780.68,3432.48) | 5365.07 (4768.75,6083.16) | 3845.50 (3392.29,4382.53) | 6877.04 (6082.77,7758.09) | 5092.90 (4445.30,5814.81) | 4008.41 (3505.92,4592.30) | 6241.49 (5437.44,7132.64) | 3816.50 (3403.01,4276.61) | 2887.23 (2571.44,3247.74) | 4735.13 (4217.91,5314.64) |
| 2001 | 2459.58 (2205.97,2723.73) | 1880.21 (1687.54,2081.88) | 3055.02 (2749.85,3384.84) | 5377.40 (4778.54,6092.09) | 3845.83 (3395.19,4373.26) | 6903.11 (6110.35,7779.92) | 5119.89 (4463.96,5852.80) | 4053.02 (3541.37,4635.99) | 6246.40 (5429.33,7150.65) | 3816.11 (3401.81,4276.97) | 2890.19 (2571.88,3251.68) | 4731.22 (4210.90,5312.10) |
| 2002 | 2446.39 (2194.74,2703.02) | 1873.06 (1681.40,2071.84) | 3034.11 (2727.24,3352.72) | 5373.42 (4771.56,6079.20) | 3829.94 (3387.60,4344.64) | 6912.69 (6128.79,7789.40) | 5173.25 (4500.57,5920.55) | 4120.12 (3598.36,4708.45) | 6282.15 (5453.25,7206.04) | 3819.67 (3403.25,4281.97) | 2896.58 (2575.35,3260.25) | 4731.91 (4207.04,5314.85) |
| 2003 | 2437.45 (2192.97,2692.64) | 1869.09 (1679.04,2066.62) | 3018.71 (2714.19,3337.15) | 5362.45 (4758.73,6055.87) | 3807.58 (3365.48,4309.68) | 6914.64 (6128.79,7790.52) | 5233.03 (4544.84,5997.25) | 4190.73 (3657.25,4783.30) | 6327.72 (5503.85,7272.60) | 3824.19 (3405.11,4288.21) | 2903.50 (2581.72,3268.43) | 4733.98 (4206.53,5315.62) |
| 2004 | 2426.93 (2183.74,2680.04) | 1863.44 (1674.62,2059.37) | 3002.23 (2697.56,3313.87) | 5353.87 (4749.56,6034.98) | 3788.48 (3348.33,4285.96) | 6917.82 (6131.42,7795.10) | 5279.19 (4589.50,6056.60) | 4245.75 (3703.03,4840.43) | 6362.07 (5539.60,7321.85) | 3826.76 (3405.03,4292.29) | 2908.22 (2584.53,3272.44) | 4734.34 (4205.75,5320.23) |
| 2005 | 2409.43 (2168.57,2659.14) | 1851.32 (1663.61,2045.96) | 2978.34 (2681.73,3284.79) | 5357.06 (4749.63,6030.77) | 3782.44 (3348.31,4275.21) | 6931.19 (6122.97,7799.25) | 5291.53 (4606.12,6076.27) | 4265.97 (3716.14,4858.80) | 6364.05 (5532.29,7338.87) | 3824.02 (3400.85,4288.00) | 2907.60 (2582.94,3270.67) | 4729.33 (4198.14,5318.89) |
| 2006 | 2375.33 (2141.19,2618.61) | 1824.66 (1638.83,2016.42) | 2935.88 (2647.61,3239.74) | 5377.71 (4775.88,6060.31) | 3791.64 (3362.74,4288.66) | 6964.02 (6170.87,7836.45) | 5156.80 (4495.25,5893.98) | 4169.87 (3645.21,4758.81) | 6186.99 (5388.36,7093.32) | 3791.56 (3379.72,4252.95) | 2883.45 (2567.00,3244.84) | 4688.41 (4168.69,5267.57) |
| 2007 | 2327.10 (2098.92,2570.62) | 1785.24 (1604.86,1974.61) | 2878.04 (2600.33,3173.84) | 5409.19 (4812.56,6100.58) | 3808.00 (3373.60,4305.47) | 7011.33 (6231.03,7904.53) | 4851.20 (4239.36,5523.91) | 3945.43 (3467.98,4496.18) | 5794.46 (5073.28,6603.61) | 3722.93 (3327.60,4172.13) | 2831.68 (2522.14,3180.21) | 4602.84 (4103.06,5166.78) |
| 2008 | 2277.34 (2054.96,2516.98) | 1743.05 (1570.39,1928.65) | 2819.91 (2552.57,3110.55) | 5442.48 (4841.09,6136.19) | 3825.96 (3393.95,4334.76) | 7060.56 (6271.74,7947.04) | 4488.18 (3957.55,5083.86) | 3677.59 (3251.74,4162.55) | 5329.81 (4686.60,6048.71) | 3642.85 (3258.09,4079.36) | 2771.15 (2469.21,3107.15) | 4503.02 (4023.21,5041.81) |
| 2009 | 2238.36 (2023.79,2474.43) | 1707.99 (1536.71,1886.94) | 2776.46 (2511.65,3062.57) | 5468.42 (4869.22,6172.83) | 3839.92 (3405.98,4354.84) | 7098.89 (6299.84,8000.67) | 4180.34 (3699.57,4707.96) | 3450.71 (3055.57,3879.57) | 4935.30 (4363.11,5575.42) | 3575.67 (3208.83,3998.73) | 2720.37 (2425.46,3049.69) | 4419.18 (3952.07,4944.97) |
| 2010 | 2222.71 (2009.23,2455.53) | 1689.93 (1520.99,1873.73) | 2762.81 (2500.81,3043.03) | 5477.79 (4875.34,6193.59) | 3844.30 (3409.64,4363.47) | 7113.46 (6326.14,8034.09) | 4039.63 (3586.44,4545.60) | 3348.77 (2974.36,3774.48) | 4752.26 (4208.96,5355.88) | 3545.42 (3176.63,3959.77) | 2697.63 (2405.79,3025.47) | 4381.19 (3922.95,4894.12) |
| 2011 | 2234.30 (2016.98,2467.12) | 1688.27 (1518.75,1869.12) | 2787.47 (2519.95,3078.14) | 5436.34 (4846.19,6139.04) | 3813.76 (3382.46,4326.25) | 7061.13 (6281.56,7967.89) | 4024.40 (3567.93,4526.17) | 3341.82 (2967.26,3759.89) | 4726.87 (4186.88,5321.83) | 3544.55 (3179.56,3966.05) | 2697.83 (2406.49,3025.03) | 4379.24 (3909.31,4901.31) |
| 2012 | 2261.88 (2039.08,2500.58) | 1693.32 (1521.08,1875.80) | 2837.53 (2559.28,3134.97) | 5339.11 (4758.02,6024.04) | 3743.36 (3318.78,4239.02) | 6937.01 (6172.36,7820.17) | 4016.95 (3553.13,4515.52) | 3341.99 (2968.34,3752.40) | 4710.06 (4165.17,5300.88) | 3547.70 (3170.81,3975.93) | 2701.80 (2408.35,3031.41) | 4381.70 (3905.95,4912.56) |
| 2013 | 2295.07 (2063.11,2536.06) | 1701.32 (1525.65,1879.90) | 2895.85 (2604.27,3202.48) | 5220.65 (4647.18,5885.19) | 3657.98 (3247.07,4135.50) | 6785.35 (6039.76,7645.15) | 4014.40 (3552.33,4517.67) | 3346.05 (2968.41,3756.61) | 4699.35 (4157.44,5289.86) | 3553.50 (3164.11,3985.40) | 2708.06 (2412.91,3040.20) | 4387.15 (3902.42,4930.35) |
| 2014 | 2323.27 (2084.22,2568.55) | 1708.51 (1529.83,1890.50) | 2945.21 (2642.73,3268.57) | 5115.44 (4549.29,5760.08) | 3582.44 (3183.40,4043.90) | 6650.37 (5919.99,7490.15) | 4013.65 (3552.72,4519.66) | 3350.63 (2966.96,3766.36) | 4691.99 (4144.89,5282.65) | 3560.51 (3165.22,4001.72) | 2715.18 (2420.07,3051.47) | 4394.14 (3893.94,4945.81) |
| 2015 | 2336.27 (2092.63,2587.11) | 1711.17 (1528.90,1899.20) | 2968.58 (2656.37,3300.51) | 5058.08 (4490.02,5690.02) | 3541.62 (3137.85,3992.99) | 6576.40 (5854.91,7400.45) | 4011.30 (3550.28,4519.00) | 3352.03 (2969.29,3764.43) | 4685.09 (4134.37,5276.53) | 3567.05 (3161.45,4011.90) | 2721.65 (2419.45,3055.58) | 4400.94 (3893.96,4960.28) |
| 2016 | 2332.96 (2087.13,2589.66) | 1708.33 (1526.09,1891.25) | 2964.17 (2659.05,3287.96) | 5044.65 (4488.20,5697.31) | 3533.66 (3136.34,3987.39) | 6557.44 (5842.25,7404.84) | 4008.52 (3546.82,4510.19) | 3351.23 (2972.10,3761.48) | 4679.89 (4130.13,5268.46) | 3570.87 (3167.45,4018.62) | 2725.50 (2418.24,3063.05) | 4405.02 (3897.66,4967.51) |
| 2017 | 2327.71 (2080.40,2581.95) | 1705.20 (1521.78,1895.01) | 2956.26 (2649.85,3281.23) | 5039.43 (4491.93,5701.15) | 3531.17 (3134.59,3989.01) | 6549.37 (5830.80,7410.89) | 4005.26 (3543.20,4501.73) | 3349.75 (2972.06,3761.68) | 4674.63 (4118.95,5261.55) | 3574.94 (3173.33,4020.79) | 2729.87 (2422.30,3071.08) | 4409.13 (3908.48,4967.91) |
| 2018 | 2318.64 (2075.89,2578.45) | 1699.43 (1520.69,1892.05) | 2944.38 (2625.77,3279.75) | 5039.54 (4468.67,5697.68) | 3533.57 (3126.46,3992.60) | 6546.86 (5811.93,7410.07) | 3994.27 (3528.41,4513.20) | 3344.82 (2956.41,3759.88) | 4657.26 (4114.50,5257.19) | 3581.29 (3165.74,4034.91) | 2738.45 (2425.41,3084.81) | 4413.67 (3894.66,4981.14) |
| 2019 | 2301.41 (2051.04,2570.60) | 1688.81 (1505.21,1886.11) | 2922.07 (2598.40,3271.13) | 5047.93 (4448.85,5704.27) | 3544.57 (3122.70,4021.21) | 6552.24 (5771.95,7430.87) | 3975.68 (3497.85,4501.39) | 3336.93 (2940.70,3757.38) | 4627.55 (4071.64,5239.88) | 3588.25 (3152.71,4060.42) | 2750.27 (2419.66,3104.07) | 4416.34 (3886.90,5015.49) |

**Table S3** Age-based variation in age-standardized incidence rates for depressive disorders in China, the United States, India and the global, 1990 to 2019

| Year | Age | Incidence (per 100,000) | | | |
| --- | --- | --- | --- | --- | --- |
|  |  | China | the United States | India | Global |
| 1994 | 20 - 24 | 3674.94 | 7859.97 | 5822.94 | 4820.91 |
|  | 25 - 29 | 3556.83 | 7367.13 | 6666.10 | 4797.95 |
|  | 30 - 34 | 3594.89 | 6863.42 | 7391.22 | 4998.22 |
|  | 35 - 39 | 3835.12 | 6294.21 | 7984.93 | 5291.68 |
|  | 40 - 44 | 4047.59 | 5779.92 | 8334.82 | 5495.38 |
|  | 45 - 49 | 4170.45 | 5320.50 | 8474.52 | 5650.71 |
|  | 50 - 54 | 4279.40 | 4863.91 | 8643.59 | 5791.33 |
|  | 55 - 59 | 4407.37 | 4414.44 | 8872.60 | 5932.06 |
|  | 60 - 64 | 4484.69 | 4213.69 | 8747.07 | 5964.45 |
|  | 65 - 69 | 4531.78 | 4266.76 | 8312.53 | 5910.40 |
|  | 70 - 74 | 4610.59 | 4308.80 | 7922.46 | 5838.95 |
|  | 75 - 79 | 4679.65 | 4353.56 | 7471.90 | 5790.76 |
|  | 80 - 84 | 4782.50 | 4524.92 | 6951.79 | 5861.10 |
|  | 85 - 89 | 4988.69 | 4830.33 | 6429.14 | 6066.98 |
| 1999 | 20 - 24 | 3035.95 | 9225.46 | 5349.98 | 4768.35 |
|  | 25 - 29 | 2942.51 | 8353.20 | 6215.37 | 4678.96 |
|  | 30 - 34 | 3065.07 | 7753.45 | 7068.03 | 4832.69 |
|  | 35 - 39 | 3346.62 | 7448.98 | 7899.71 | 5300.69 |
|  | 40 - 44 | 3569.04 | 6921.51 | 8342.27 | 5544.49 |
|  | 45 - 49 | 3772.75 | 6231.68 | 8567.20 | 5650.19 |
|  | 50 - 54 | 3956.02 | 5524.01 | 8798.28 | 5797.70 |
|  | 55 - 59 | 4115.80 | 4819.70 | 9060.64 | 6000.28 |
|  | 60 - 64 | 4223.21 | 4464.25 | 8994.16 | 6061.14 |
|  | 65 - 69 | 4259.46 | 4466.14 | 8443.40 | 5921.57 |
|  | 70 - 74 | 4299.20 | 4467.46 | 7871.00 | 5827.92 |
|  | 75 - 79 | 4378.14 | 4471.60 | 7317.34 | 5692.64 |
|  | 80 - 84 | 4511.59 | 4519.43 | 6853.92 | 5690.16 |
|  | 85 - 89 | 4714.00 | 4636.85 | 6442.98 | 5882.52 |
| 2004 | 20 - 24 | 2671.92 | 9173.42 | 5482.85 | 4765.47 |
|  | 25 - 29 | 2597.19 | 8073.98 | 6245.44 | 4716.66 |
|  | 30 - 34 | 2714.23 | 7568.07 | 7053.62 | 4812.34 |
|  | 35 - 39 | 3030.19 | 7660.27 | 7930.50 | 5178.39 |
|  | 40 - 44 | 3300.42 | 7320.21 | 8481.82 | 5535.91 |
|  | 45 - 49 | 3560.62 | 6534.55 | 8792.99 | 5676.04 |
|  | 50 - 54 | 3858.93 | 5736.80 | 9203.11 | 5774.73 |
|  | 55 - 59 | 4144.47 | 4924.46 | 9598.75 | 5972.77 |
|  | 60 - 64 | 4289.18 | 4475.78 | 9576.85 | 6107.98 |
|  | 65 - 69 | 4370.90 | 4395.75 | 9091.81 | 6017.65 |
|  | 70 - 74 | 4455.70 | 4313.23 | 8488.41 | 5846.87 |
|  | 75 - 79 | 4560.60 | 4238.65 | 7859.74 | 5715.56 |
|  | 80 - 84 | 4719.85 | 4222.12 | 7379.69 | 5634.37 |
|  | 85 - 89 | 4915.46 | 4291.61 | 7007.87 | 5731.22 |
| 2009 | 20 - 24 | 2168.69 | 9293.44 | 3944.03 | 4205.41 |
|  | 25 - 29 | 2134.41 | 8101.30 | 4192.89 | 4217.85 |
|  | 30 - 34 | 2266.05 | 7660.83 | 4778.11 | 4360.82 |
|  | 35 - 39 | 2566.40 | 7994.41 | 5707.38 | 4706.66 |
|  | 40 - 44 | 2940.21 | 7696.27 | 6424.49 | 4990.79 |
|  | 45 - 49 | 3351.35 | 6814.46 | 6945.00 | 5299.89 |
|  | 50 - 54 | 3760.48 | 5906.31 | 7484.84 | 5504.02 |
|  | 55 - 59 | 4194.76 | 4985.42 | 8075.03 | 5692.79 |
|  | 60 - 64 | 4452.25 | 4452.28 | 8290.42 | 5835.03 |
|  | 65 - 69 | 4544.83 | 4341.99 | 8166.48 | 5873.96 |
|  | 70 - 74 | 4664.96 | 4224.82 | 8023.42 | 5857.22 |
|  | 75 - 79 | 4813.75 | 4108.80 | 7793.52 | 5768.45 |
|  | 80 - 84 | 4994.54 | 4081.13 | 7421.44 | 5749.19 |
|  | 85 - 89 | 5257.34 | 4155.70 | 6903.70 | 5787.77 |
| 2014 | 20 - 24 | 2131.53 | 8802.95 | 3746.08 | 4212.73 |
|  | 25 - 29 | 2095.91 | 7560.84 | 3961.76 | 4094.32 |
|  | 30 - 34 | 2211.99 | 6956.29 | 4485.80 | 4346.39 |
|  | 35 - 39 | 2495.85 | 6994.76 | 5318.92 | 4721.55 |
|  | 40 - 44 | 2931.08 | 6695.09 | 6012.89 | 4940.86 |
|  | 45 - 49 | 3525.94 | 6067.97 | 6584.62 | 5166.84 |
|  | 50 - 54 | 4078.80 | 5457.10 | 7157.98 | 5476.30 |
|  | 55 - 59 | 4620.91 | 4840.10 | 7737.21 | 5717.53 |
|  | 60 - 64 | 4976.87 | 4461.15 | 8045.70 | 5842.87 |
|  | 65 - 69 | 5100.10 | 4320.85 | 8013.07 | 5850.26 |
|  | 70 - 74 | 5208.12 | 4182.07 | 8002.77 | 5877.29 |
|  | 75 - 79 | 5363.38 | 4038.96 | 7969.75 | 5873.68 |
|  | 80 - 84 | 5509.59 | 3986.52 | 7649.98 | 5817.62 |
|  | 85 - 89 | 5698.24 | 4053.63 | 7093.42 | 5861.02 |
| 2019 | 20 - 24 | 2153.63 | 8907.79 | 3625.95 | 4298.45 |
|  | 25 - 29 | 2150.48 | 7812.77 | 3936.17 | 4202.56 |
|  | 30 - 34 | 2256.45 | 6995.93 | 4480.09 | 4320.40 |
|  | 35 - 39 | 2472.69 | 6463.77 | 5264.02 | 4777.93 |
|  | 40 - 44 | 2865.10 | 5993.39 | 5935.81 | 5046.65 |
|  | 45 - 49 | 3425.63 | 5605.42 | 6519.15 | 5207.22 |
|  | 50 - 54 | 4002.11 | 5208.33 | 7112.01 | 5434.40 |
|  | 55 - 59 | 4529.08 | 4832.33 | 7698.98 | 5741.98 |
|  | 60 - 64 | 4852.10 | 4576.54 | 7993.07 | 5867.02 |
|  | 65 - 69 | 5027.43 | 4449.79 | 7999.16 | 5853.26 |
|  | 70 - 74 | 5189.53 | 4308.29 | 7983.09 | 5840.99 |
|  | 75 - 79 | 5348.92 | 4173.37 | 7989.36 | 5855.51 |
|  | 80 - 84 | 5540.67 | 4122.39 | 7740.46 | 5878.51 |
|  | 85 - 89 | 5799.83 | 4189.37 | 7181.40 | 5917.55 |

**Table S4** Cohort-based variation in age-standardized incidence rates for depressive disorders in China, the United States, India and the global, 1990 to 2019

| Age | Cohort | Incidence (per 100,000) | | | |
| --- | --- | --- | --- | --- | --- |
|  |  | China | the United States | India | Global |
| 20 - 24 | 1974 | 4578.36 | 9960.46 | 5822.94 | 4820.91 |
|  | 1979 | 3889.89 | 11599.40 | 5349.98 | 4768.35 |
|  | 1984 | 3488.32 | 11508.67 | 5482.85 | 4765.47 |
|  | 1989 | 3015.51 | 11551.27 | 3944.03 | 4205.41 |
|  | 1994 | 3018.67 | 10898.41 | 3746.08 | 4212.73 |
|  | 1999 | 3073.33 | 11108.27 | 3625.95 | 4298.45 |
| 25 - 29 | 1969 | 4364.66 | 9087.64 | 6666.10 | 4797.95 |
|  | 1974 | 3720.50 | 10354.64 | 6215.37 | 4678.96 |
|  | 1979 | 3322.68 | 10025.66 | 6245.44 | 4716.66 |
|  | 1984 | 2880.14 | 9961.44 | 4192.89 | 4217.85 |
|  | 1989 | 2871.20 | 9286.53 | 3961.76 | 4094.32 |
|  | 1994 | 2937.33 | 9632.30 | 3936.17 | 4202.56 |
| 30 - 34 | 1964 | 4322.98 | 8297.98 | 7391.22 | 4998.22 |
|  | 1969 | 3790.54 | 9480.51 | 7068.03 | 4832.69 |
|  | 1974 | 3378.37 | 9235.76 | 7053.62 | 4812.34 |
|  | 1979 | 2943.73 | 9224.45 | 4778.11 | 4360.82 |
|  | 1984 | 2904.23 | 8396.84 | 4485.80 | 4346.39 |
|  | 1989 | 2958.55 | 8523.14 | 4480.09 | 4320.40 |
| 35 - 39 | 1959 | 4501.14 | 7541.34 | 7984.93 | 5291.68 |
|  | 1964 | 4034.24 | 9000.10 | 7899.71 | 5300.69 |
|  | 1969 | 3661.54 | 9146.09 | 7930.50 | 5178.39 |
|  | 1974 | 3207.72 | 9368.08 | 5707.38 | 4706.66 |
|  | 1979 | 3139.83 | 8240.59 | 5318.92 | 4721.55 |
|  | 1984 | 3125.71 | 7794.26 | 5264.02 | 4777.93 |
| 40 - 44 | 1954 | 4682.32 | 6867.91 | 8334.82 | 5495.38 |
|  | 1959 | 4226.12 | 8277.59 | 8342.27 | 5544.49 |
|  | 1964 | 3903.87 | 8612.26 | 8481.82 | 5535.91 |
|  | 1969 | 3559.78 | 8892.89 | 6424.49 | 4990.79 |
|  | 1974 | 3536.20 | 7774.83 | 6012.89 | 4940.86 |
|  | 1979 | 3485.73 | 7152.34 | 5935.81 | 5046.65 |
| 45 - 49 | 1949 | 4795.33 | 6279.98 | 8474.52 | 5650.71 |
|  | 1954 | 4416.98 | 7380.26 | 8567.20 | 5650.19 |
|  | 1959 | 4149.00 | 7624.96 | 8792.99 | 5676.04 |
|  | 1964 | 3951.29 | 7851.61 | 6945.00 | 5299.89 |
|  | 1969 | 4103.54 | 7012.52 | 6584.62 | 5166.84 |
|  | 1974 | 4030.20 | 6620.52 | 6519.15 | 5207.22 |
| 50 - 54 | 1944 | 4896.77 | 5699.93 | 8643.59 | 5791.33 |
|  | 1949 | 4585.57 | 6473.45 | 8798.28 | 5797.70 |
|  | 1954 | 4440.94 | 6632.14 | 9203.11 | 5774.73 |
|  | 1959 | 4342.96 | 6789.73 | 7484.84 | 5504.02 |
|  | 1964 | 4625.15 | 6274.20 | 7157.98 | 5476.30 |
|  | 1969 | 4593.87 | 6085.95 | 7112.01 | 5434.40 |
| 55 - 59 | 1939 | 5018.90 | 5130.31 | 8872.60 | 5932.06 |
|  | 1944 | 4732.14 | 5573.59 | 9060.64 | 6000.28 |
|  | 1949 | 4718.39 | 5631.62 | 9598.75 | 5972.77 |
|  | 1954 | 4766.94 | 5721.30 | 8075.03 | 5692.79 |
|  | 1959 | 5138.12 | 5535.17 | 7737.21 | 5717.53 |
|  | 1964 | 5103.96 | 5577.02 | 7698.98 | 5741.98 |
| 60 - 64 | 1934 | 5052.67 | 4817.40 | 8747.07 | 5964.45 |
|  | 1939 | 4793.83 | 5069.27 | 8994.16 | 6061.14 |
|  | 1944 | 4826.41 | 5041.85 | 9576.85 | 6107.98 |
|  | 1949 | 4986.30 | 5061.89 | 8290.42 | 5835.03 |
|  | 1954 | 5458.26 | 5043.94 | 8045.70 | 5842.87 |
|  | 1959 | 5383.49 | 5199.17 | 7993.07 | 5867.02 |
| 65 - 69 | 1929 | 5020.18 | 4765.16 | 8312.53 | 5910.40 |
|  | 1934 | 4752.49 | 4967.28 | 8443.40 | 5921.57 |
|  | 1939 | 4843.59 | 4868.10 | 9091.81 | 6017.65 |
|  | 1944 | 5014.87 | 4846.21 | 8166.48 | 5873.96 |
|  | 1949 | 5532.93 | 4803.62 | 8013.07 | 5850.26 |
|  | 1954 | 5490.98 | 4961.79 | 7999.16 | 5853.26 |
| 70 - 74 | 1924 | 5019.99 | 4700.48 | 7922.46 | 5838.95 |
|  | 1929 | 4715.25 | 4861.35 | 7871.00 | 5827.92 |
|  | 1934 | 4863.43 | 4689.12 | 8488.41 | 5846.87 |
|  | 1939 | 5070.58 | 4621.45 | 8023.42 | 5857.22 |
|  | 1944 | 5591.17 | 4562.99 | 8002.77 | 5877.29 |
|  | 1949 | 5583.30 | 4707.96 | 7983.09 | 5840.99 |
| 75 - 79 | 1919 | 5007.73 | 4636.50 | 7471.90 | 5790.76 |
|  | 1924 | 4716.39 | 4755.34 | 7317.34 | 5692.64 |
|  | 1929 | 4902.19 | 4514.96 | 7859.74 | 5715.56 |
|  | 1934 | 5153.72 | 4395.52 | 7793.52 | 5768.45 |
|  | 1939 | 5696.52 | 4315.83 | 7969.75 | 5873.68 |
|  | 1944 | 5671.51 | 4458.79 | 7989.36 | 5855.51 |
| 80 - 84 | 1914 | 5034.34 | 4724.79 | 6951.79 | 5861.10 |
|  | 1919 | 4773.04 | 4719.30 | 6853.92 | 5690.16 |
|  | 1924 | 4989.39 | 4420.14 | 7379.69 | 5634.37 |
|  | 1929 | 5263.16 | 4283.63 | 7421.44 | 5749.19 |
|  | 1934 | 5777.91 | 4183.35 | 7649.98 | 5817.62 |
|  | 1939 | 5792.15 | 4322.63 | 7740.46 | 5878.51 |
| 85 - 89 | 1909 | 5171.18 | 4975.45 | 6429.14 | 6066.98 |
|  | 1914 | 4903.65 | 4781.94 | 6442.98 | 5882.52 |
|  | 1919 | 5109.43 | 4435.34 | 7007.87 | 5731.22 |
|  | 1924 | 5450.91 | 4302.82 | 6903.70 | 5787.77 |
|  | 1929 | 5890.55 | 4196.54 | 7093.42 | 5861.02 |
|  | 1934 | 5981.27 | 4335.05 | 7181.40 | 5917.55 |

**Table S5** Age–period–cohort (APC) model analysis results of depressive disorders incidence in China, the United States, India and global, by gender.

| Variables | China  (Coef, 95% CI) | | | the United States  (Coef, 95% CI) | | | India  (Coef, 95% CI) | | | Global  (Coef, 95% CI) | | |
| --- | --- | --- | --- | --- | --- | --- | --- | --- | --- | --- | --- | --- |
|  | both | males | females | both | males | females | both | males | females | both | males | females |
| Age |  |  |  |  |  |  |  |  |  |  |  |  |
| 20-24 | -0.06*** (-0.08,-0.04) | -0.06*** (-0.08,-0.04) | -0.05*** (-0.07,-0.04) | 0.33*** (0.32,0.34) | 0.33*** (0.32,0.34) | 0.36*** (0.35,0.37) | -0.18*** (-0.19,-0.17) | -0.23*** (-0.25,-0.22) | -0.14*** (-0.15,-0.13) | -0.09*** (-0.10,-0.08) | -0.09*** (-0.11,-0.08) | -0.08*** (-0.09,-0.06) |
| 25-29 | -0.19*** (-0.20,-0.17) | -0.15*** (-0.17,-0.14) | -0.19*** (-0.21,-0.18) | 0.23*** (0.22,0.24) | 0.25*** (0.24,0.26) | 0.24*** (0.24,0.25) | -0.15*** (-0.16,-0.14) | -0.18*** (-0.19,-0.17) | -0.13*** (-0.14,-0.12) | -0.13*** (-0.14,-0.11) | -0.12*** (-0.13,-0.11) | -0.11*** (-0.13,-0.10) |
| 30-34 | -0.24*** (-0.26,-0.23) | -0.21*** (-0.23,-0.19) | -0.25*** (-0.26,-0.24) | 0.17*** (0.16,0.18) | 0.20*** (0.19,0.21) | 0.18*** (0.17,0.19) | -0.10*** (-0.11,-0.09) | -0.12*** (-0.13,-0.11) | -0.09*** (-0.10,-0.08) | -0.12*** (-0.13,-0.10) | -0.11*** (-0.12,-0.10) | -0.10*** (-0.11,-0.09) |
| 35-39 | -0.23*** (-0.25,-0.22) | -0.22*** (-0.24,-0.20) | -0.23*** (-0.24,-0.21) | 0.17*** (0.16,0.18) | 0.17*** (0.16,0.19) | 0.18*** (0.17,0.19) | -0.04*** (-0.05,-0.03) | -0.05*** (-0.06,-0.04) | -0.03*** (-0.04,-0.02) | -0.06*** (-0.07,-0.05) | -0.06*** (-0.08,-0.05) | -0.04*** (-0.05,-0.03) |
| 40-44 | -0.20*** (-0.22,-0.19) | -0.20*** (-0.22,-0.18) | -0.18*** (-0.20,-0.17) | 0.13*** (0.12,0.14) | 0.12*** (0.11,0.13) | 0.15*** (0.14,0.16) | -0.01** (-0.02,-0.01) | -0.02** (-0.03,-0.01) | 0.00 (-0.01,0.01) | -0.03*** (-0.04,-0.01) | -0.03*** (-0.05,-0.02) | 0.00 (-0.01,0.01) |
| 45-49 | -0.15*** (-0.16,-0.13) | -0.15*** (-0.17,-0.14) | -0.12*** (-0.14,-0.11) | 0.05*** (0.04,0.06) | 0.03*** (0.02,0.05) | 0.08*** (0.07,0.09) | 0.00 (-0.01,0.01) | -0.01* (-0.02,0.00) | 0.02*** (0.01,0.03) | -0.01 (-0.02,0.01) | -0.02** (-0.03,-0.01) | 0.02*** (0.01,0.03) |
| 50-54 | -0.08*** (-0.09,-0.07) | -0.10*** (-0.11,-0.08) | -0.05*** (-0.06,-0.04) | -0.03*** (-0.04,-0.02) | -0.06*** (-0.07,-0.05) | 0.00 (-0.01,0.01) | 0.02*** (0.01,0.03) | 0.01 (0.00,0.02) | 0.05*** (0.04,0.06) | 0.02** (0.00,0.03) | -0.01 (-0.02,0.01) | 0.04*** (0.03,0.05) |
| 55-59 | 0.00 (-0.01,0.01) | -0.03** (-0.04,-0.01) | 0.03*** (0.02,0.04) | -0.12*** (-0.13,-0.11) | -0.17*** (-0.18,-0.15) | -0.09*** (-0.10,-0.08) | 0.07*** (0.06,0.08) | 0.04*** (0.03,0.05) | 0.09*** (0.08,0.10) | 0.04*** (0.03,0.06) | 0.02* (0.00,0.03) | 0.07*** (0.06,0.08) |
| 60-64 | 0.06*** (0.04,0.07) | 0.03*** (0.02,0.05) | 0.08*** (0.07,0.09) | -0.16*** (-0.18,-0.15) | -0.20*** (-0.22,-0.19) | -0.14*** (-0.15,-0.13) | 0.08*** (0.07,0.09) | 0.06*** (0.05,0.07) | 0.10*** (0.09,0.10) | 0.06*** (0.05,0.07) | 0.04*** (0.03,0.05) | 0.08*** (0.07,0.09) |
| 65-69 | 0.10*** (0.08,0.11) | 0.08*** (0.07,0.10) | 0.11*** (0.10,0.12) | -0.15*** (-0.16,-0.14) | -0.16*** (-0.17,-0.14) | -0.15*** (-0.16,-0.14) | 0.07*** (0.06,0.08) | 0.07*** (0.06,0.08) | 0.06*** (0.06,0.07) | 0.06*** (0.05,0.07) | 0.06*** (0.04,0.07) | 0.06*** (0.05,0.07) |
| 70-74 | 0.15*** (0.14,0.16) | 0.14*** (0.13,0.15) | 0.15*** (0.14,0.16) | -0.15*** (-0.16,-0.13) | -0.12*** (-0.14,-0.11) | -0.17*** (-0.18,-0.16) | 0.07*** (0.06,0.07) | 0.09*** (0.08,0.10) | 0.04*** (0.03,0.05) | 0.05*** (0.04,0.07) | 0.07*** (0.06,0.08) | 0.04*** (0.03,0.05) |
| 75-79 | 0.21*** (0.20,0.22) | 0.21*** (0.19,0.22) | 0.19*** (0.18,0.20) | -0.15*** (-0.17,-0.14) | -0.10*** (-0.12,-0.09) | -0.20*** (-0.21,-0.19) | 0.07*** (0.06,0.08) | 0.12*** (0.11,0.13) | 0.02*** (0.01,0.03) | 0.05*** (0.04,0.06) | 0.08*** (0.07,0.09) | 0.01** (0.00,0.02) |
| 80-84 | 0.28*** (0.27,0.29) | 0.28*** (0.27,0.30) | 0.24*** (0.22,0.25) | -0.16*** (-0.17,-0.15) | -0.12*** (-0.13,-0.10) | -0.22*** (-0.23,-0.21) | 0.06*** (0.06,0.07) | 0.12*** (0.11,0.13) | 0.01* (0.00,0.02) | 0.06*** (0.05,0.07) | 0.09*** (0.07,0.10) | 0.01 (0.00,0.02) |
| 85-89 | 0.36*** (0.35,0.38) | 0.37*** (0.35,0.38) | 0.28*** (0.26,0.29) | -0.16*** (-0.17,-0.15) | -0.18*** (-0.19,-0.16) | -0.22*** (-0.23,-0.21) | 0.05*** (0.04,0.06) | 0.10*** (0.09,0.11) | 0.00 (-0.01,0.01) | 0.08*** (0.07,0.09) | 0.09*** (0.08,0.10) | 0.01* (0.00,0.02) |
| Period |  |  |  |  |  |  |  |  |  |  |  |  |
| 1994 | 0.07*** (0.06,0.08) | 0.08*** (0.07,0.09) | 0.07*** (0.06,0.08) | -0.03*** (-0.03,-0.02) | -0.04*** (-0.05,-0.03) | -0.02*** (-0.03,-0.02) | 0.07*** (0.06,0.07) | 0.00 (0.00,0.01) | 0.12*** (0.11,0.12) | 0.02*** (0.01,0.02) | 0.00 (0.00,0.01) | 0.03*** (0.02,0.03) |
| 1999 | -0.03*** (-0.04,-0.02) | -0.01** (-0.02,-0.01) | -0.04*** (-0.05,-0.03) | 0.07*** (0.06,0.07) | 0.07*** (0.06,0.08) | 0.06*** (0.06,0.07) | 0.05*** (0.05,0.06) | 0.03*** (0.02,0.03) | 0.07*** (0.07,0.08) | 0.01*** (0.01,0.02) | 0.01* (0.00,0.02) | 0.02*** (0.01,0.02) |
| 2004 | -0.05*** (-0.06,-0.04) | -0.03*** (-0.04,-0.02) | -0.06*** (-0.07,-0.05) | 0.05*** (0.04,0.06) | 0.05*** (0.04,0.06) | 0.05*** (0.04,0.06) | 0.10*** (0.09,0.10) | 0.10*** (0.10,0.11) | 0.09*** (0.09,0.10) | 0.02*** (0.01,0.02) | 0.02*** (0.01,0.03) | 0.02*** (0.01,0.02) |
| 2009 | -0.06*** (-0.07,-0.05) | -0.05*** (-0.06,-0.05) | -0.07*** (-0.07,-0.06) | 0.04*** (0.04,0.05) | 0.04*** (0.04,0.05) | 0.04*** (0.04,0.05) | -0.07*** (-0.08,-0.07) | -0.05*** (-0.05,-0.04) | -0.09*** (-0.10,-0.09) | -0.03*** (-0.03,-0.02) | -0.02*** (-0.03,-0.02) | -0.03*** (-0.03,-0.02) |
| 2014 | 0.03*** (0.02,0.03) | 0.00 (-0.01,0.01) | 0.04*** (0.03,0.05) | -0.05*** (-0.05,-0.04) | -0.04*** (-0.05,-0.04) | -0.05*** (-0.05,-0.04) | -0.08*** (-0.09,-0.08) | -0.05*** (-0.06,-0.05) | -0.11*** (-0.11,-0.10) | -0.02*** (-0.03,-0.01) | -0.01*** (-0.02,-0.01) | -0.02*** (-0.03,-0.02) |
| 2019 | 0.04*** (0.04,0.05) | 0.02*** (0.01,0.03) | 0.05*** (0.05,0.06) | -0.08*** (-0.09,-0.08) | -0.08*** (-0.09,-0.07) | -0.09*** (-0.09,-0.08) | -0.06*** (-0.07,-0.06) | -0.03*** (-0.04,-0.02) | -0.09*** (-0.09,-0.08) | 0.00 (-0.01,0.00) | 0.01 (0.00,0.01) | -0.01** (-0.02,0.00) |
| Cohort |  |  |  |  |  |  |  |  |  |  |  |  |
| 1909 | -0.09*** (-0.12,-0.06) | -0.11*** (-0.14,-0.08) | -0.10*** (-0.13,-0.07) | 0.04** (0.01,0.06) | 0.08*** (0.05,0.11) | -0.01 (-0.04,0.01) | -0.14*** (-0.16,-0.12) | -0.07*** (-0.09,-0.04) | -0.19*** (-0.21,-0.17) | 0.04** (0.01,0.06) | 0.04** (0.01,0.07) | 0.01 (-0.01,0.03) |
| 1914 | -0.05*** (-0.07,-0.03) | -0.07*** (-0.09,-0.04) | -0.05*** (-0.07,-0.04) | -0.07*** (-0.09,-0.05) | -0.07*** (-0.09,-0.04) | -0.09*** (-0.11,-0.08) | -0.10*** (-0.12,-0.08) | -0.06*** (-0.07,-0.04) | -0.13*** (-0.15,-0.12) | 0.02 (0.00,0.03) | 0.01 (-0.01,0.03) | -0.01 (-0.02,0.01) |
| 1919 | 0.00 (-0.01,0.02) | -0.02 (-0.03,0.00) | 0.00 (-0.02,0.01) | -0.12*** (-0.14,-0.10) | -0.14*** (-0.16,-0.12) | -0.13*** (-0.15,-0.12) | -0.06*** (-0.07,-0.04) | -0.04*** (-0.06,-0.03) | -0.07*** (-0.08,-0.06) | 0.00 (-0.02,0.01) | 0.01 (-0.01,0.02) | -0.02** (-0.03,-0.01) |
| 1924 | 0.06*** (0.05,0.07) | 0.05*** (0.03,0.06) | 0.05*** (0.04,0.07) | -0.15*** (-0.17,-0.14) | -0.17*** (-0.19,-0.15) | -0.15*** (-0.17,-0.14) | 0.01* (0.00,0.02) | 0.02** (0.01,0.03) | 0.01 (0.00,0.02) | 0.01 (0.00,0.02) | 0.01 (-0.01,0.02) | 0.00 (-0.01,0.01) |
| 1929 | 0.09*** (0.08,0.11) | 0.10*** (0.08,0.11) | 0.09*** (0.08,0.10) | -0.15*** (-0.17,-0.14) | -0.17*** (-0.19,-0.15) | -0.15*** (-0.16,-0.14) | 0.08*** (0.07,0.09) | 0.07*** (0.06,0.08) | 0.08*** (0.07,0.09) | 0.03*** (0.02,0.04) | 0.03*** (0.02,0.04) | 0.03*** (0.01,0.04) |
| 1934 | 0.12*** (0.11,0.14) | 0.14*** (0.12,0.15) | 0.12*** (0.11,0.13) | -0.14*** (-0.15,-0.12) | -0.15*** (-0.16,-0.13) | -0.13*** (-0.14,-0.12) | 0.13*** (0.12,0.14) | 0.11*** (0.10,0.12) | 0.14*** (0.13,0.15) | 0.04*** (0.03,0.05) | 0.04*** (0.03,0.06) | 0.04*** (0.03,0.05) |
| 1939 | 0.16*** (0.15,0.17) | 0.18*** (0.17,0.20) | 0.16*** (0.15,0.17) | -0.13*** (-0.14,-0.11) | -0.13*** (-0.15,-0.12) | -0.12*** (-0.13,-0.11) | 0.17*** (0.17,0.18) | 0.15*** (0.14,0.16) | 0.18*** (0.17,0.19) | 0.06*** (0.04,0.07) | 0.06*** (0.05,0.07) | 0.06*** (0.05,0.07) |
| 1944 | 0.19*** (0.18,0.21) | 0.21*** (0.20,0.23) | 0.19*** (0.18,0.20) | -0.10*** (-0.12,-0.09) | -0.10*** (-0.12,-0.09) | -0.10*** (-0.11,-0.09) | 0.20*** (0.19,0.21) | 0.17*** (0.16,0.18) | 0.21*** (0.20,0.22) | 0.06*** (0.05,0.07) | 0.07*** (0.05,0.08) | 0.06*** (0.05,0.08) |
| 1949 | 0.22*** (0.21,0.24) | 0.24*** (0.22,0.25) | 0.22*** (0.21,0.23) | -0.07*** (-0.08,-0.06) | -0.06*** (-0.07,-0.04) | -0.07*** (-0.08,-0.06) | 0.20*** (0.19,0.21) | 0.18*** (0.17,0.19) | 0.22*** (0.21,0.23) | 0.05*** (0.04,0.07) | 0.06*** (0.05,0.07) | 0.06*** (0.05,0.07) |
| 1954 | 0.24*** (0.22,0.25) | 0.24*** (0.23,0.26) | 0.23*** (0.22,0.25) | -0.03*** (-0.04,-0.02) | -0.01 (-0.03,0.00) | -0.03*** (-0.04,-0.02) | 0.20*** (0.19,0.21) | 0.18*** (0.17,0.19) | 0.21*** (0.20,0.22) | 0.05*** (0.04,0.06) | 0.05*** (0.04,0.07) | 0.05*** (0.04,0.06) |
| 1959 | 0.22*** (0.21,0.24) | 0.23*** (0.21,0.24) | 0.23*** (0.21,0.24) | 0.02** (0.01,0.03) | 0.04*** (0.03,0.05) | 0.01** (0.00,0.03) | 0.18*** (0.17,0.19) | 0.16*** (0.15,0.17) | 0.19*** (0.18,0.20) | 0.05*** (0.04,0.06) | 0.05*** (0.04,0.06) | 0.05*** (0.04,0.06) |
| 1964 | 0.19*** (0.17,0.20) | 0.18*** (0.16,0.20) | 0.20*** (0.18,0.21) | 0.06*** (0.05,0.07) | 0.09*** (0.07,0.10) | 0.06*** (0.05,0.07) | 0.15*** (0.14,0.16) | 0.13*** (0.12,0.14) | 0.16*** (0.15,0.17) | 0.04*** (0.03,0.05) | 0.04*** (0.02,0.05) | 0.04*** (0.03,0.06) |
| 1969 | 0.12*** (0.11,0.14) | 0.11*** (0.09,0.12) | 0.13*** (0.12,0.15) | 0.08*** (0.07,0.09) | 0.10*** (0.09,0.11) | 0.08*** (0.07,0.09) | 0.10*** (0.09,0.11) | 0.08*** (0.07,0.09) | 0.11*** (0.10,0.12) | 0.01 (-0.01,0.02) | 0.00 (-0.02,0.01) | 0.01* (0.00,0.02) |
| 1974 | 0.02** (0.01,0.04) | 0.00 (-0.02,0.02) | 0.04*** (0.03,0.05) | 0.08*** (0.07,0.09) | 0.09*** (0.08,0.10) | 0.08*** (0.07,0.09) | 0.02*** (0.01,0.03) | 0.00 (-0.01,0.01) | 0.03*** (0.03,0.04) | -0.02** (-0.03,-0.01) | -0.03*** (-0.04,-0.02) | -0.01 (-0.02,0.00) |
| 1979 | -0.09*** (-0.11,-0.07) | -0.11*** (-0.13,-0.09) | -0.08*** (-0.09,-0.06) | 0.08*** (0.07,0.09) | 0.08*** (0.07,0.09) | 0.08*** (0.08,0.09) | -0.07*** (-0.08,-0.06) | -0.09*** (-0.10,-0.08) | -0.05*** (-0.06,-0.04) | -0.03*** (-0.04,-0.02) | -0.04*** (-0.06,-0.03) | -0.02*** (-0.03,-0.01) |
| 1984 | -0.21*** (-0.23,-0.19) | -0.21*** (-0.23,-0.19) | -0.21*** (-0.22,-0.19) | 0.09*** (0.08,0.10) | 0.08*** (0.07,0.09) | 0.10*** (0.09,0.11) | -0.16*** (-0.17,-0.15) | -0.17*** (-0.19,-0.16) | -0.15*** (-0.16,-0.14) | -0.05*** (-0.07,-0.04) | -0.06*** (-0.08,-0.04) | -0.05*** (-0.06,-0.03) |
| 1989 | -0.33*** (-0.35,-0.30) | -0.31*** (-0.34,-0.28) | -0.34*** (-0.36,-0.32) | 0.12*** (0.11,0.14) | 0.10 (0.09,0.12) | 0.14*** (0.13,0.15) | -0.25*** (-0.27,-0.24) | -0.24*** (-0.26,-0.22) | -0.26*** (-0.27,-0.24) | -0.11*** (-0.12,-0.09) | -0.10*** (-0.12,-0.08) | -0.10*** (-0.12,-0.09) |
| 1994 | -0.41*** (-0.44,-0.39) | -0.39*** (-0.43,-0.36) | -0.42*** (-0.44,-0.40) | 0.17*** (0.16,0.19) | 0.15 (0.13,0.17) | 0.19*** (0.18,0.20) | -0.31*** (-0.33,-0.29) | -0.28*** (-0.30,-0.25) | -0.31*** (-0.33,-0.30) | -0.11*** (-0.13,-0.09) | -0.11*** (-0.13,-0.09) | -0.11*** (-0.13,-0.09) |
| 1999 | -0.48*** (-0.52,-0.43) | -0.46*** (-0.51,-0.41) | -0.47*** (-0.51,-0.43) | 0.21*** (0.19,0.24) | 0.20 (0.17,0.22) | 0.23*** (0.21,0.25) | -0.36*** (-0.39,-0.32) | -0.32*** (-0.36,-0.29) | -0.37*** (-0.40,-0.34) | -0.12*** (-0.15,-0.08) | -0.11*** (-0.15,-0.08) | -0.11*** (-0.14,-0.08) |
| AIC | 13.26 | 11.72 | 15.06 | 16.33 | 14.36 | 18.47 | 17.20 | 15.37 | 20.29 | 11.91 | 11.43 | 12.29 |
| BIC | -17.12 | -123.55 | 116.40 | 208.53 | 69.29 | 370.62 | 261.83 | 124.61 | 507.38 | -160.60 | -178.32 | -144.82 |
| Deviance | 195.56 | 89.12 | 329.08 | 421.21 | 281.97 | 583.30 | 474.51 | 337.29 | 720.06 | 52.08 | 34.36 | 67.86 |

Note: * p < 0.05, ** p < 0.01, *** p < 0.001. Coef, coefficient; AIC, Akaike’s information criterion; BIC, Bayesian information criterion.
